# High-resolution analysis of red deer (*Cervus elaphus*) management units in a Central European region of high human population density reveals severe effects on genetic diversity and differentiation

**DOI:** 10.1371/journal.pone.0327427

**Published:** 2025-06-27

**Authors:** Julian Laumeier, Corinna Klein, Hermann Willems, Gerald Reiner

**Affiliations:** Department for Veterinary Clinical Science and Work Group Wildlife Biology, Justus Liebig University, Giessen, Germany; Texas State University, UNITED STATES OF AMERICA

## Abstract

The threat of isolation to red deer (*Cervus elaphus*) has been described in numerous European studies. The consequences range from reduced genetic diversity and increased inbreeding to inbreeding depression. It has been shown that the underlying factors cannot be generalised, but vary greatly in their effects depending on local conditions. The aim of this study was to analyse in detail the genetics of red deer in a large German federal state with a population density of 532 inhabitants per km^2^ and 23.8% settlement and traffic area, in order to generate data for future management of the region. 1199 individual samples of red deer were collected in all 20 Administrative Management Units (AMUs) and compared with existing results from the neighbouring state of Hesse (19 AMUs). All 2490 individuals from both states were clustered using Bayesian methods and connectivity between neighbouring AMUs was quantified. Overall, 30% of the AMUs were found to be highly isolated, mostly with effective population sizes (Ne) < 100. In contrast, 47.5% of the AMUs still had clear connectivity, allowing them to be merged into 4 larger red deer regions. For the small isolated areas, low genetic diversity was found in units with high homozygosity and low Ne. With high sampling density and identical methodology, detailed information on AMUs can be obtained and the degree of vulnerability of individual AMUs as part of the overall population can specifically be validated. Such data can help improve future wildlife management.

## Introduction

In Central Europe, red deer (*Cervus elaphus*) can potentially colonise extensive areas, usually in considerable numbers [1]. Although originally adapted to semi-open landscapes [2,3], they are predominantly pushed back into forest structures as part of management concepts [4,5]. This highly migratory species would indeed move between summer habitats at higher altitudes and winter habitats in the lowlands [6]. However, such migrations are now prevented in many places by dense settlement [7], especially in former floodplains, and by anthropogenic barriers such as transport infrastructure [8,9]. This isolation has led to significant genetic differentiation, especially in central European populations [10]. Favourable numbers of animals and the presence of suitable habitats are of little use in terms of the ability of species to survive if populations are isolated from each other [11,12]. In some areas, alarming effective population sizes and levels of inbreeding have already been identified [13–15]. Of the 20 European populations sampled by Zachos et al. [10], 18 did not meet the internationally accepted effective population size of 1000 for long-term adaptive populations [16]. In 50% of the populations analysed, the effective population size was less than 100. According to Frankham et al. [16], such populations can no longer respond independently to inbreeding depression. Studies from Spain [17], Scotland and England [18,19], Denmark [20] and Sweden [21] confirm this assumption based on local populations.

Similarly, random samples of German red deer populations have been analysed, confirming that they are also highly differentiated [22]. However, given the migratory behaviour of red deer, which according to Peters et al. [6] covers *radii* of approximately 21 km and an average of 28 km for young males [23], gene flow can be expected to follow the stepping stone principle [24], so that successive habitats and populations can be expected to have naturally decreasing gene flow with distance [25,26]. Accordingly, isolated populations can only be identified by considering all local populations and migration opportunities, as shown by Kuehn et al. [27] for Bavaria and Reiner et al. [28] for Hesse. For example, Edelhoff et al. [29] found that in Schleswig-Holstein, where red deer are on the IUCN Red List of Threatened Species, none of the twelve populations reached an effective population size of 1000 and only 25% of the populations were above 100. At the same time, different regions in the above studies showed considerable differences in population density, anthropogenic infrastructure and the natural distribution of both forest habitat and red deer. The management of red deer in different regions also varies considerably, particularly depending on local hunting laws. While in the north-east of Germany red deer are theoretically free to roam depending on infrastructure, in the south-west red deer areas are designated as Administrative Management Units (AMUs) and red deer must be hunted strictly outside these areas to prevent their establishment and spread and to protect forests from damage.

The effects of such anthropogenic boundaries on genetic characteristics are known for some red deer populations in Europe and especially in Germany [28,29]. However, these studies do not reveal a general, overarching and easily applicable principle. Rather, they show considerable variation in the effects of the factors and the need for detailed analysis for individual regions.

The aim of the present study was to provide the first meaningful population genetic comparison of all red deer populations in two directly neighbouring federal states which, despite some geographical and landscape similarities, differ in terms of anthropogenic characteristics and red deer management [30–34]. This study was conducted using previously tested markers [28,35,36] to ensure maximum comparability. It was hypothesised that significant qualitative and quantitative differences in genetic diversity and differentiation between the red deer populations of the two states occur due to considerable anthropogenic differences between Hesse (19 red deer populations, 303 inhabitants/km^2^, 40% forest, 16.2% settlement and traffic area) and North Rhine-Westphalia (NRW) (21 red deer areas, 532 inhabitants/km^2^, 24.8% forest, 23.8% settlement and traffic area).

## Materials and methods

### Ethical statement

No permits were required for the present study, because no animal experiments were performed. Samples of red deer previously shot by authorised persons during legalised hunting were used. No animals were killed specifically for the study. No living animals were sampled and no dropping antlers were sought or collected for the study.

### Study area, red deer populations and sampling

The study area covers the entire region of the federal states of Hesse and North Rhine-Westphalia (NRW) in central and western Germany with a north-south extension of 350 km, a west-east extension of 290 km and a total area of approximately 55,213 km^2^.

Compared to the German average of 237 persons per km^2^, the population density in 2023 was about 531.7 persons per km^2^ in North Rhine-Westphalia and 302.6 persons per km^2^ in Hesse [37]. Hesse consists of different types of land use, mainly forests (42.5%), pastures (13.4%) and agriculture (22.6%). Hesse is the German state with the largest forest area. The corresponding land use proportions for forest, pasture and agriculture in NRW were 26.9%, 12.1% and 31.1%, respectively.

**Fig 1:**
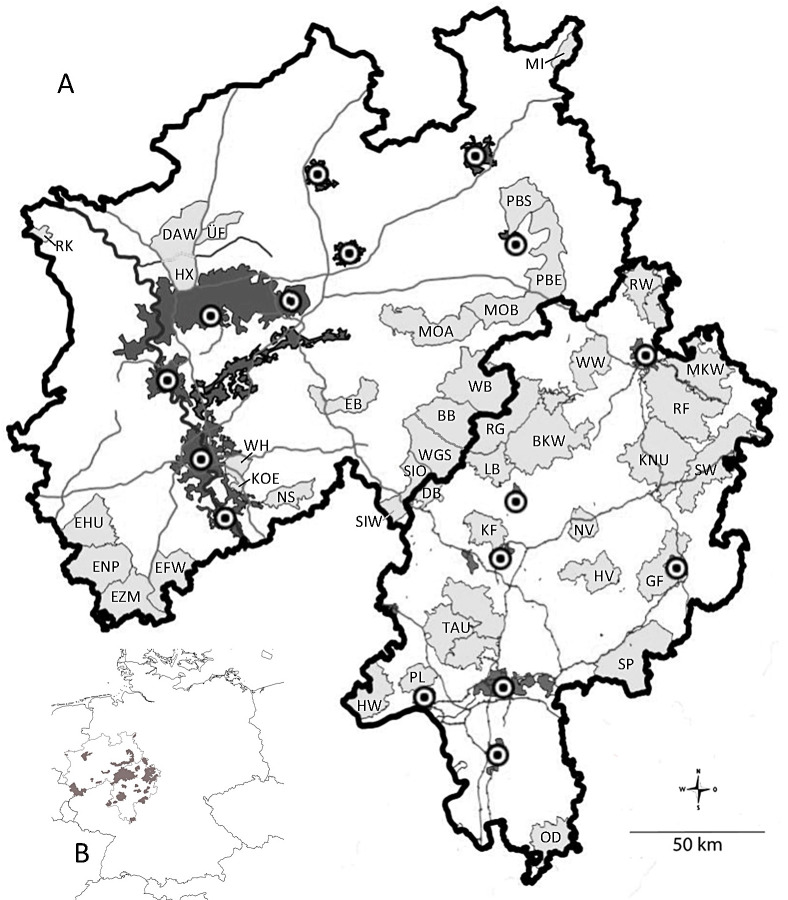
Geographical location of the German federal states surveyed and location and extent of the 40 AMUs surveyed within the study area. All existing AMUs were sampled. Dark grey areas: settlement areas; grey lines: motorways; light grey areas: AMUs. Description of the abbreviations and details of the AMUs are given in Table 1A and 1B.

The AMUs are scattered across the two states and vary considerably in size from 41.3 (RK) to 787.2 km^2^ (TAU) (Fig 1, Tables 1A and 1B). Together, 40 AMUs were surveyed. The distances between AMUs ranged from 7.64 (DB-SIO) to 321.41 km (OD-RK) (centre to centre). They were separated by residential areas, fenced motorways, country roads or by larger contiguous agricultural areas. In the ‘red deer–free’ areas between AMUs, establishment of red deer populations should be prevented to protect vegetation from damage. Hunting in these areas could impede the migration of red deer between AMUs [38]. According to the local authorities and the red deer management associations legally responsible for the management of the respective AMUs, the estimated population size of the AMUs in spring ranged from 59 animals in Wattenberg-Weidelsburg (WW) to 3480 animals in Eifel-Zitterwald-Mürrel (EZM) (Table 1). However, these figures are based on estimates, as exact numbers are not available. The estimates are based on calculations of hunting bag numbers according to the official federal processes of the states Hesse and Northrhine-Westphalia [39–43].

**Table 1:** A. Description of the red deer administrative management units (AMUs) in North Rhine-Westphalia (NRW). B. Description of the red deer administrative management units (AMUs) in Hesse.

A													
Number	Region^*^	Subregion^*^	AMU^*^	Abbreviation	AMU size (km^2^)	Estimated population		Animals/ km^2^	Samples	Latitude	Longitude	Extension N-S (km)	Extension W-O (km)
**1**	Eifel			EI	1343.7	8217		6.1	353	50.56951	6.54187	54.80	56.26
**1.1**			Venn – Hürtgenwald	EHU	299.4	869		2.9	96	50.71814	6.31983	21.71	24.75
**1.2**			Nord- und Rureifel (Nationalpark)	ENP	378.6	2340		6.2	147	50.53526	6.37232	22.36	28.14
**1.3**			Zitterwald – Mürel	EZM	375.5	3488		9.3	48	50.43791	6.57194	25.51	32.57
**1.4**			Flamersheimer Wald	EFW	290.2	1520		5.2	62	50.49371	6.79625	27.12	20.25
**2**	Reichswald Kleve		Reichswald Kleve	RK	41.3	146		3.5	19	51.74323	6.03374	6.49	11.28
**3**	Dämmerwald – Herrlichkeit Lembeck			NR	545.3	1349		2.5	162	51.69768	6.93478	35.70	36.97
**3.1**			Hünxe	HX	140.8	348		2.5	104	51.40584	6.84093	41.19	13.83
**3.2**			Üfter Mark – Dämmerwald^**^	UF (DAW)^*^	404.5	1001		2.5	58	51.75157	6.93478	23.70	36.95
**4**	Wahner Heide		Wahner Heide – Königsforst^***^	WH (KOE)^**^	94.1	340		3.6	69	50.88551	7.17506	15.94	11.23
**5**	Nutscheid		Nutscheid	NS	130.7	67		0.5	12	50.81674	7.45952	12.37	21.53
**6**	Ebbegebirge		Ebbegebirge	EB	194.6	159		0.8	29	51.16981	7.77236	14.93	28.03
**7**	Möhnetal			MT	658.3	1089		1.7	145	51.43993	8.46499	21.02	67.25
**7.1**			Arnsberger Wald	MOA	377.1	681		1.8	91	51.42033	8.26779	16.66	39.93
**7.2**			Brilon – Büren	MOB	281.2	408		1.5	54	51.47040	8.69824	14.24	34.88
**8**	Eggegebirge – Teutoburger Wald – Senne			ETS	586.4	1227		2.1	146	51.72470	8.85997	48.34	26.73
**8.1**			Eggegebirge	PBE	470.4	984		2.1	86	51.68505	8.92978	39.51	17.13
**8.2**			Senne	PBS	116.0	243		2.1	60	51.83965	8.80862	22.75	19.59
**9**	Minden		Minden	MI	254.9	163		0.6	34	52.38159	9.10599	23.13	19.60
**10**	Rothaargebirge			RHG	2711.3	8549		3.2	457	51.02486	8.58477	71.29	86.98
**10.1**		Olpe-Siegerland		SI	202.4	1495		7.4	89	50.83764	8.12392	29.62	22.54
**10.1.1**			Siegen West	SIW	79.8	590		7.4	36	50.76050	8.05809	12.45	13.30
**10.1.2**			Siegen Ost	SIO	122.6	905		7.4	53	50.87741	8.18805	20.76	13.52
**10.2**		Wittgenstein-Schmallenberg		WSS	651.0	2102		3.2	91	51.01313	8.33078	33.82	30.48
**10.2.1**			Wittgenstein	WGS	357.9	1155		3.2	48	50.97493	8.30408	25.37	22.40
**10.2.2**			Bad Berleburg	BB	293.2	947		3.2	43	51.06734	8.33210	21.57	30.47
**10.3**			Rothaargebirge Nord (Winterberg)	WB	483.3	1681		3.5	50	51.22019	8.53848	27.81	31.98
			Total	NRW (AMU’s)	6305.7	18080		2.9	1199	51.40442	7.60104	240.65	229.20
**B**													
**Number**	Region^*^	Subregion^*^	AMU^*^	Abbrevi-ation	AMU size (km^2^)		Estimated population	Animals/ km^2^	Samples	Latitude	Longitude	Extension N-S (km)	Extension W-O (km)
**10**	Rothaargebirge												
**10.4**			Dill-Bergland	DB	109.3		1146	10.5	57	50.81799	8.24233	14.37	15.80
**10.5**		Rothaargebirge und Lahnbergland		RGL	599.1		1259	2.1	114	51.03365	8.67817	44.65	31.77
**10.5.1**			Rothaargebirge	RG	410.2		862	2.1	54	51.09129	8.68952	31.82	30.17
**10.5.2**			Lahn-Bergland	LB	188.9		397	2.1	60	50.91430	8.60156	18.08	21.11
**10.6**			Burgwald-Kellerwald	BKW	644.0		785	1.2	56	51.03382	8.94773	34.79	36.18
**11**	Wattenberg-Weidelsburg		Wattenberg-Weidelsburg	WW	239.4		59	0.2	59	51.28276	9.18325	22.99	16.60
**12**	Reinhardswald		Reinhardswald	RW	314.5		681	2.2	204	51.52944	9.50616	28.45	18.96
**13**	Waldhessen			NH	1950.3		6864	3.5	269	51.06327	9.73429	72.32	55.57
**13.1**			Meißner-Kaufunger Wald	MKW	384.7		1742	4.5	78	51.26068	9.81034	28.38	27.90
**13.2**			Riedforst	RF	559.0		2117	3.8	60	51.14505	9.71642	33.76	34.18
**13.3**			Knüllwald	KNU	555.8		1364	2.5	56	50.93559	9.56631	33.83	32.08
**13.4**			Seulingswald	SW	450.8		1641	3.6	75	50.91868	9.90227	40.14	32.11
**14**	Vogelsberg			VB	618.8		1399	2.3	155	50.58804	9.38074	38.58	54.06
**14.1**			Gieseler Forst	GF	292.0		407	1.4	53	50.54960	9.60547	30.02	22.30
**14.2**			Hoher Vogelsberg	HV	214.0		795	3.7	47	50.52412	9.17757	17.27	25.39
**14.3**			Nördlicher Vogelsberg	NV	112.9		197	1.7	55	50.69844	9.14238	14.00	12.17
**15**	Spessart		Spessart	SP	467.8		2741	5.9	73	50.21670	9.43522	29.26	32.84
**16**	Krofdorfer Forst		Krofdorfer Forst	KF	171.6		158	0.9	59	50.67109	8.58510	17.05	17.77
**17**	Rheingau-Taunus			RGT	1165.3		5346	4.6	185	50.24703	8.23438	57.44	65.67
**17.1**			Taunus	TAU	787.2		2623	3.3	69	50.33699	8.45854	37.41	33.63
**17.2**			Platte	PL	121.6		243	2	48	50.14053	8.19863	11.50	15.95
**17.3**			Hinterlandswald	HW	256.4		2480	9.7	68	50.09859	7.91772	24.39	20.59
**18**	Odenwald		Odenwald	OD	246.1		1832	7.4	60	49.53840	8.97384	22.05	21.58
			**Total**	**Hesse (AMU’s)**	**6543.4**		**22351**	**3.4**	**1291**	**50.54829**	**8.95227**	**246.85**	**167.04**

AMU: administrative management unit;

* : Some regions were divided into subregions, with some of these regions and subregions containing several AMUs. All AMUs are shown in Fig 1;

** : There were only 9 samples from DAW which were included into UF for analysis;

*** : There were only 4 samples from KOE which were included into WH for analysis. AMU: administrative management unit;

Samples from red deer of both sexes were collected during legal hunting activities. The tissues were taken from skeletal muscle, spleen or lung of freshly shot animals. The main purpose of the hunts was to manage the red deer population and for human consumption of the meat. Samples were labelled with information on the animal (sex and age class) and the hunting area, and frozen until processed in the laboratory. A total of 1199 samples were collected in NRW during the 2020–2022 hunting seasons. No animals were killed specifically for the study. No live animals were sampled and no shed antlers were sought or collected for the study. Due to the impact of sample size on the accuracy of population genetic results [35], the goal was to collect 60 samples per area. In the end, an average of 63.8 samples were collected per area (0.194 individuals per km^2^). Only a few populations had less than 60 samples because the number of animals in the hunt was not sufficient. The minimum sample size was 12 samples in Nutscheid (see Table 1A and B for details). AMUs with close geographical/political links were grouped together to form ‘larger regions’, e.g., the Eifel (EI) with the individual AMUs EHU, ENP, EZM and EFW (see Fig 1 and Table 1A).

No new samples were collected for Hesse. Rather, data from the already genotyped samples from 1291 individuals collected during the 2018/2019 hunting season (Fig 1, Table 1B) [28] were used and analysed together with the newly collected and genotyped samples from NRW. In total, samples from 2490 red deer individuals were analysed.

### DNA extraction and genotyping

DNA was extracted using a commercially available kit (Instant Virus RNA Kit, Analytik Jena, Germany). For this purpose, 30–50 mg of tissue was processed according to the manufacturer’s instructions. DNA concentration was determined photometrically and adjusted to 5 ng/μl with RNAse-free water. The presence of high molecular weight DNA was confirmed by agarose gel electrophoresis. Sixteen microsatellites were used to genotype red deer (Table 2). The same markers were used and combined in two multiplex PCRs as by Willems et al. (2016) and Reiner et al., 2021 [28,36]. PCR was performed in a volume of 10 μl consisting of 5 μl 2 × multiplex mastermix (Qiagen, Germany), 4 μl primer mix and 1 μl (5 ng) extracted DNA. After an initial denaturation step of 15 min, the DNA was amplified in 26 cycles of denaturation at 94 °C for 30 s, annealing at 56 °C (multiplex PCR 4 at 50 °C) for 90 s and extension at 72 °C for 30 s. After a final step at 60 °C for 30 min, the PCR reactions were cooled to 4 °C.

**Table 2:** Description of the microsatellite markers used.

Marker		Primer 5’ – 3’	5’-Mod.^3^	Colour	Low^4^	High^5^	PCR^6^
**CE-BM4208**	F^1^	GGT TTT CCT CAA CTG AGT GCT	6-FAM	Y	270	330	1
	R^2^	TGG GAT CAA TCC TGA CCT TGA A					
**CE-CSSM14**	F	AGC CGT TGA AAA TGG ATA ATT C	HEX	B	120	135	1
	R	GGC TAA CAG AAG AGA AGC CTC A					
**CE-CSSM16**	F	CTC TTG GTT TCA AAG AGC CAC T	Atto550	B	138	172	1
	R	CTT GAT TCA AAT GTC AGG CAA A					
**CE-CSSM19**	F	GGG AAA CTA ACA CAG GGT CTG A	6-FAM	G	150	195	1
	R	AGA AAA CAC AAG TGG GAA GTG C					
**CE-CSSM22**	F	AAC AGT CAA GAT TCT GGG AGG A	6-FAM	B	173	193	1
	R	AGA GTC TGC GCT CTT ACC AAC T					
							
**ETH225**	F	AAG AGC AAT TGG GGA TTA TCT G	HEX	B	222	265	1
	R	CAG CAA GAA AGA GAA GGT ATG GA					
**Haut14**	F	TGT GAT CCC AAC AAG TAA CAC A	HEX	Y	180	220	1
	R	TAC CAG GGA AGA TGA AAT GAC C					
**T501**	F	CTA AGC CTG CTC ACT GCT ACA A	6-FAM	G	210	265	1
	R	TGG ATG AGG ATA CTA AGG CTC A					
**BM1818**	F	CAG CTG GGA ATA TAA CCA AAG G	HEX	B	210	250	2
	R	GGT CCA GCA TGT TAT CAC AAA					
**INRA35**	F	TGT CTT CTC AGG CTG ATT TCT G	Atto550	B	100	145	2
	R2	CAT CTC CAA CTA CTA GGA AAC AGA C					
**MM12**	F	TCC AGC AAG ACA GGT GTT TCA A	6-FAM	G	125	150	2
	R	TCT CTT TAG GGC AGA TGG GAT C					
**NVHRT48**	F	TCT TTG TCT AGC TGT TCC GTG A	6-FAM	Y	130	210	2
	R	CCC ATC AGT TTG TCT CCT GAA G					
**NVHRT73**	F	GAT GGA TAT GAC TTG CCC ATT T	6-FAM	G	250	295	2
	R	TAA CAG GGA GAA GTG GGG TCT A					
**RT1**	F	AGC TTG CCT TAA GTG TCT GCT T	6-FAM	B	260	310	2
	R	TGT GAT CAT GCC TTC TTT CAT C					
**RT6**	F	GTG GCA ACA AGA CTG CAA TTA G	Atto550	G	180	230	2
	R	CTG GGC AGA GTG ATT CCT CTT A					
**T172**	F	TTG ATC TAG CAT CTC CCC TTT C	HEX	B	147	205	2
	R	CAA GTA TCG AAC CCG TAT CTC C					

^1^: forward primer;

^2^: reverse primer;

^3^: dye modification (Y = yellow colour; B = blue colour; G = green colour;

^4^: shortest allel;

^5^: longest allel;

^6^: number of multiplex PCR.

### Capillary electrophoresis

One microliter of fluorescently labelled PCR product and 0.375 µl DNA Size Standard 500 Orange (Nimagen, The Netherlands) were added to 12 µl Hi-Di-formamide (Thermofisher Scientific, Germany) and electrophoresed on an ABI PRISM 310 automated sequencer. Allele sizes were determined using Peakscanner 2.0 software (Thermofisher Scientific, Germany).

### Analyses of population genetic parameters

Most of the population genetic analyses were performed in the statistical software R, Version 4.2.1 [44]. Null allele frequencies were calculated using the null.all function implemented in the R package PopGenReport v3.0.4 [45]. As the frequency of missing data was less than 5%, null allele frequencies were estimated using the method described by Brookfield [46]. The 95% confidence interval (CI) was calculated using 1,000 bootstraps. If the 95% CI includes zero, the null allele frequencies are not significantly different from zero. Deviations from Hardy-Weinberg equilibrium (HWE) were tested with the function hw.test implemented in the R package pegas v0.12 [47]. The test was performed as an exact test based on Monte Carlo permutations (n = 1,000) of alleles [48].

Private alleles were determined using functions implemented in the R package poppr v2.8.3 [49]. The absolute number of alleles (An), the allelic richness (Ar), the average number of alleles per marker (Na), the expected (He) and observed (Ho) heterozygosity and the inbreeding coefficient according to Wright were calculated as standard population genetic parameters using the divBasic function implemented in the R-package diveRsity V. 1.9.90 (Keenan et al. [50]. Fis values were reported after 1,000 bootstrap iterations. [51]

The R package diveRsity v.1.9.90 [50] was used to determine pairwise population differentiation using Fst [52] and Jost’s D [53] as metrics. Significance of differences in pairwise comparisons was assessed by 1,000 bootstrapping iterations. Fst calculations were compared with and without null alleles using FreeNA software [54]. While Fst reflects demographic processes and fixation, Jost’s D is a measure of allelic differentiation [55].

Effective population size (Ne) was estimated using NeEstimator V2.1 [56]. Estimates of Ne were calculated using the linkage disequilibrium method with random mating as the mating system. To exclude single-copy alleles, the critical value for allele frequency (Pcrit) was set to 0.02 for populations with less than 50 individuals sampled, and to 0.01 for populations with 50 or more individuals sampled. Additionally, for comparison, Ne was determined from demographic data provided by local authorities and local red deer conservation societies, who predetermine the hunting of males and females at equal shares by law [30–34]. Assuming a constant sex ratio of reproducing animals and no fluctuations in population size according to Willems et al. [36], Ne was calculated from the number of reproducing males (Nm) and females (Nf) according to Wang et al. [57] under the assumption of harem polygamy as a mode of reproduction as Ne=(4*Nm*Nf)/(2*Nm + Nf). In addition, the formula of Caballero “under lottery polygyny” Ne=(4mfN)/(1+(m/n)) was also used (m = % of males; f = % of females; N = population size; n = Lifetime breeding success of females) [58].

To compare the AMUs, the allelic potential (Na(p)) was calculated as a new parameter to estimate the number of alleles depending on the actual population size of the AMUs given above. The allelic potential was based on the formula described by Reiner et al. [35]. Na(p) = found allels * (1/(13.69ln(x)+38.685), where x is the percentage of sampled individuals in the population.

Isolation by distance was checked using the “mantel” function of the “vegan” package, Version 2.6–6.1 [59]. According to Loe et al. [23], who showed an average movement of 28 km for young deer, only neighbouring AMU’s within a maximum distance of 40 km were used so that the average distance of AMU’s (average 28.2 km; centre to centre) corresponded to the realistic movement of young deer. Associations between geographical distance and genetic distance are shown graphically. The correlation of this function was used to show excessive and limited connectivity between neighbouring AMUs. Additionally the estimated effective Migration Surface (EEMS) was analysed following the instructions of Petkova et al. [60]. For the calculations we estimated 660 demes to build triangles with a side length of about 20 km, which corresponds to red deer moving distances. The further values were set to negBiProb = 0.5, mseedsProposalS2 = 0.1, qEffctProposalS2 = 0.01, all other values were set to default.

To assess the genetic structure of the population, we used STRUCTURE 2.3.4 [61–63], DAPC, using the R-Package “adegenet” Version 1.3−1 [64], BAPS, Version 6.0, [65] and TESS 2.3 [66,67]. STRUCTURE uses a Bayesian model-based clustering method with a heuristic approach to estimate the number of clusters (K). The STRUCTURE analysis was performed with K = 1–50 clusters, assuming admixture and correlated allele frequencies. For each K, 10 independent runs with 100,000 burn-in and 200,000 MCMC iterations were performed. The optimal number of K was determined by the method of Evanno et al. [68] using the R-package “pophelper”, version 2.3.1 [69]. The most likely K-value was only recognised as a crude measure to describe the level of genetic structure patterns. To identify underlying nested clusters, a hierarchical STRUCTURE analysis was performed [29], using the clusters from previous runs as input and setting ‘LOCPRIOR’ as the sampling location. Subsequent analyses were performed only on the clusters identified in the previous run. According to Gilbert [70], this approach was repeated until (1) all samples came from the same sampling location, (2) the average assignment of individuals in the previous run fell below 80%, or (3) fewer than ten animals remained in a cluster.

Population structure was additionally assessed using the clustering procedure of Discriminant Analysis of Principal Components (DAPC), implemented in the R package adegenet, version 1.3−1, [64]. In a first step, a partitioning analysis with K = 2–50 was performed to determine the optimal number of clusters.

Assuming an island model, the improvement in fit was monitored by the Bayesian Information Criterion (BIC) value, which decreases until it reaches the optimal K. The results of the K-means procedure were used as input data for DAPC. The number of retained principal components was validated using the cross-validation function xvalDapc. The frequencies of individuals belonging to the different clusters were determined for each AMU.

BAPS was run with 38 populations for spatial clustering of individuals and set as admixture on mixture clustering. Other settings include 5 as a minimum size of population, 1,000 iterations, 100 reference individuals for each population, and 20 iterations for reference individual.

To estimate the number of clusters with TESS, “Total number of sweeps” was set to 1,200 and “Burn in number of sweeps” was set to 200. 2–50 clusters with ten runs each were checked for their BIC to find the optimal one, which was re-run a hundred times with a “Total Number of Sweeps” of 50,000 and a “Burn IN Number of Sweeps” of 10,000 to find the best distribution.

To quantify the genetic similarity between neighbouring AMUs, the results of DAPC alone as well as the combined results of DAPC, Structure, BAPS and TESS were converted into percentages expressing the probability that individuals from two AMUs belonged to the same cluster [28]. The average percentage was called genetic connectivity and was calculated for all pairs of AMUs.

AMUs with connectivity were combined as red deer regions if at least two of the following conditions were met: Connectivity by STRUCTURE analysis above 40, connectivity by DAPC analysis above 40, and Fst values below 0.05. This is because the population genetic distinctiveness of populations is not defined by their technical AMU classification, but by actual exchange between AMUs within a region, even if populations are assigned to different AMUs. These three methods were chosen because they are among the most commonly used methods and because they showed the highest level of agreement, and thus the most consistent result, when all methods were compared.

Accordingly, Ne and Na(p) were also calculated together for these regions based on the demographic figures, as the Ne of populations with internal substructuring and exchange would be underestimated using the Ne estimator [71].

Statements regarding IUCN criteria were made according to the formula developed by Willoughby et al. [72]: Ht = Ho(1–1/2Ne)t, where Ho is the measured heterozygosity of the population and Ht is the predicted heterozygosity in the population after t generations. Ne is the effective size of the population. Finally, t is determined as the number of generations (t) until heterozygosity falls below the 25% quantile (Ht = 0.54). Ht was determined empirically by Willoughby et al. [72] in a meta-study of 5,156 published articles on nearly 18,000 microsatellite loci in 5,900 species. As suggested by the authors, t-values ≤ 10, ≤ 50 and ≤ 100 were used as criteria for critically endangered, endangered and vulnerable populations, respectively. These t-values were applied to all populations, but a distinction was made between populations that were truly highly isolated and those with connectivity to populations of neighbouring AMUs. For populations with a high degree of isolation, all conditions for such an assessment were met, while for the remaining populations, it should be stated which criteria would be met if their connectivity could not be maintained in the future.

Statistical comparisons between regions and the two federal states were done with one-way analysis of variance in IBM-SPSS, Version 27. Mean values, standard deviations, significance levels and coefficients of determination were presented.

Maps illustrating the population genetics characteristics were constructed in QGIS, Version 3.28.3. The free and open source public software was downloaded from http://qgis.org, including the license terms (http://qgis.org/license). The authors confirm their consent for the images they have created in QGIS to be published in Plos one under a CC BY 4.0 license.

## Results

### Null alleles, private alleles, Hardy-Weinberg-equilibrium

The null allele frequencies of markers significantly different from zero ranged from 0.024% (NVHRT48, EI) to 0.298% (RT6, EFW). No instances of null alleles were observed in the AMUs BB, DAW, EB, HV, HW, MI, NS, RK, SIW and UF. The most significant deviations were observed in 20 out of 40 AMUs for RT6 (0.055% to 0.298%), followed by T172 and BM4208, which exhibited significant deviations from zero in 18 out of 40 AMUs (0.046% to 0.189%; 0.036% to 0.12%). All other deviations were evenly distributed uniformly across markers and areas. No significant differences were observed between the Fst values obtained with and without null alleles using T-test for paired samples (mean ± standard deviation without null alleles: 0.88 ± 0.47; mean ± standard deviations with null alleles: 0.86 ± 0.44; T = 1.34; P = 0.18; df = 702). The correlation between Fst values with and without null alleles was 0.71 (P = 7.63 x 10-^109^). Consequently, all loci were retained. The marker NVHRT48 exhibited the highest number of alleles (n = 40), while markers CSSM22N and CSSM14 displayed the lowest (n = 4).

A total of 32 private alleles were identified across 15 populations. The majority of private alleles were observed in the WGS and UF populations, with four alleles each. The markers with the highest number of private alleles were BM4208, CSSM19, NVHRT48 and HAUT14, with four alleles each.

The total red deer panel and the AMUs BB, EG, EHU, EI, ENP, ETS, GF, KON, MOA, MOB, MT, NH, RGT, RHG, RK, RW, SI, SIO, TAU, VB, WGS, WSS, HE, NRW exhibited a significant departure from the Hardy-Weinberg equilibrium (data not shown).

### Population genetic parameters

A total of 260 alleles were identified across the entire study area, with 250 in North Rhine-Westphalia (NRW) and 194 in Hesse (Table 3A and 3B). The NVHRT48 marker exhibited the greatest polymorphism, displaying 40 alleles. The lowest number of alleles was observed in the marker CSSM14 (n = 4), which was monomorphic in the Nutscheid (NS). Additionally, the marker CSSM22 exhibited monomorphism in both the HV and HW populations.

**Table 3:** A. Population genetic parameters of the red deer administrative management units (AMUs) in North Rhine-Westphalia (NRW). B. Population genetic parameters of the red deer administrative management units (AMUs) in Hesse.

A								
	AMU	An	Na	He	Ho	Ar	Fis	Na(p)
	NRW	250	15.6	0.80	0.69	7.76	0.136	387
**1**	EI	195	12.2	0.75	0.70	6.62	0.067	333
**1.1**	EHU	148	9.2	0.73	0.67	6.04	0.072	207
**1.2**	ENP	169	10.6	0.74	0.71	6.34	0.043	265
**1.3**	EZM	152	9.5	0.74	0.73	6.52	0.016	353
**1.4**	EFW	152	9.5	0.75	0.70	6.44	0.067	262
**2**	RK	85	5.3	0.60	0.55	4.34	0.087	115
**3**	NR	150	9.4	0.69	0.67	5.32	0.036	206
**3.1**	HX	120	7.5	0.66	0.65	4.73	0.013	151
**3.2**	UF	118	7.4	0.69	0.70	5.21	−0.018	195
**4**	WH	121	7.6	0.69	0.70	5.40	−0.010	153
**5**	NS	95	5.9	0.63	0.67	5.05	−0.060	122
**6**	EB	116	7.2	0.71	0.70	5.54	0.015	148
**7**	MT	181	11.3	0.75	0.68	6.76	0.092	244
**7.1**	MOA	153	9.6	0.72	0.66	6.19	0.078	206
**7.2**	MOB	168	10.5	0.76	0.71	6.85	0.068	227
**8**	ETS	169	10.6	0.75	0.69	6.67	0.074	233
**8.1**	EG	149	9.3	0.73	0.70	6.21	0.044	219
**8.2**	SE	122	7.6	0.71	0.69	5.75	0.027	146
**9**	MI	99	6.2	0.63	0.65	4.74	−0.034	123
**10**	RHG	208	13.0	0.74	0.69	6.68	0.068	337
**10.1**	SI	163	10.2	0.75	0.70	6.52	0.063	258
**10.1.1**	SIW	135	8.4	0.73	0.71	6.08	0.022	213
**10.1.2**	SIO	151	9.4	0.74	0.69	6.35	0.068	240
**10.2**	WSS	184	11.5	0.76	0.71	7.04	0.072	313
**10.2.1**	WGS	162	10.1	0.74	0.70	6.63	0.054	278
**10.2.2**	BB	148	9.2	0.74	0.71	6.49	0.041	249
**10.3**	WB	140	8.8	0.70	0.67	6.21	0.042	261
**B**								
	**AMU**	**An**	**Na**	**He**	**Ho**	**Ar**	**Fis**	**Na(p)**
	HE	194	12.1	0.73	0.66	6.65	0.096	309
**10.4**	DB	110	6.9	0.70	0.69	5.36	0.021	181
**10.5**	RGL	132	8.2	0.71	0.68	5.75	0.039	192
**10.5.1**	RG	127	7.9	0.70	0.68	5.76	0.032	199
**10.5.2**	LB	109	6.8	0.70	0.69	5.22	0.014	144
**10.6**	BKW	125	7.8	0.70	0.70	5.83	0.003	191
**11**	WW	123	7.7	0.70	0.69	5.52	0.026	121
**12**	RW	142	8.9	0.70	0.66	5.79	0.053	167
**13**	NH	153	9.6	0.72	0.68	6.12	0.063	267
**13.1**	MKW	112	7.0	0.68	0.66	5.26	0.029	189
**13.2**	RF	113	7.1	0.69	0.69	5.37	0.004	213
**13.3**	KNU	122	7.6	0.69	0.67	5.56	0.030	210
**13.4**	SW	127	7.9	0.68	0.69	5.57	−0.011	213

B								
**14**	VB	139	8.7	0.70	0.65	5.79	0.065	194
**14.1**	GF	107	6.7	0.66	0.62	5.35	0.071	145
**14.2**	HV	117	7.3	0.69	0.67	5.53	0.017	186
**14.3**	NV	116	7.2	0.68	0.66	5.35	0.025	138
**15**	SP	132	8.2	0.69	0.67	5.88	0.030	253
**16**	KF	106	6.6	0.67	0.65	5.20	0.032	120
**17**	RGT	141	8.8	0.68	0.64	5.44	0.054	253
**17.1**	TAU	116	7.2	0.68	0.63	5.17	0.062	223
**17.2**	PL	92	5.8	0.63	0.62	4.44	0.014	116
**17.3**	HW	111	6.9	0.65	0.66	5.10	−0.016	211
**18**	OD	105	6.6	0.64	0.62	4.88	0.034	191

AMU: Administrative Management Unit; An: Absolute number of alleles per AMU; Na: average number of alleles per marker; He: expected heterozygosity; Ho: observed heterozygosity; Fis: inbreeding coefficient according to Wright; Ar: allelic richness; Na(p): number of alleles based on estimated total population size (allel potential).

The highest number of alleles within a single AMU was observed in the ENP, with a total of 169 alleles, while the lowest number was recorded in the RK, with a total of 85 alleles (Table 3). The mean number of alleles per marker exhibited a similar pattern (maximum: ENP, 10.6; minimum: RK, 5.3). The mean number of alleles per marker was 116 in Hesse and 135 in NRW. There was a discrepancy between the allelic richness calculations produced by FSTAT and diveRsity. The results obtained from FSTAT (data not shown) consistently exceeded those yielded by diveRsity. The MOB population exhibited the highest allelic richness, with values of 6.85 (diveRsity) and 7.21 (FSTAT), respectively. The lowest allelic richness was observed in the RK (4.34 for diveRsity; 4.73 for FSTAT).

The expected heterozygosity of the entire population was 0.77, a notable discrepancy from the observed heterozygosity of 0.67 (P < 0.005). The lowest values were observed in the RK (He = 0.6; Ho = 0.55). The highest expected heterozygosity was observed in the MOB population (0.76), while the highest observed heterozygosity was noted in the EZM population in the Eifel (0.73).

The AMUs in which the proportion of homozygous loci (1-Ho) was one third or more were RK (0.45), GF, PL, OD (0.38), and TAU (0.37). The remaining populations exhibiting the highest proportion of homozygous loci were MI, HX and KF (0.35), along with the MOA, RW, MKW, NV and HW populations (0.34).

The highest value of the inbreeding coefficient Fis was observed in the RK population (0.087 diveRsity; 0.113 FSTAT), while the lowest value was recorded in the NS population (−0.06 diveRsity; −0.017 FSTAT).

In the largest region, as defined by according to Table 1A and 1B, the topographical Rothaargebirge (RHG), 457 individuals were sampled exhibiting 208 alleles (with an average of 13 alleles per marker). The Eifel (EI) region exhibited the highest expected heterozygosity (0.75) and observed heterozygosity (0.7) among the superordinate regions, indicating the presence of a relatively high level of heterozygosity. The lowest Fis value, 0.036 (FSTAT 0.039), was observed in the superior region of NR.

As the detection rate for rare alleles also increases with an increasing proportion of sampled animals, the expected allelic potential (Na(p)) was calculated for the expected population size of each AMU. The lowest allelic potential was observed in the RK with a total of 115 calculated alleles. The highest number of alleles was calculated for the AMU EZM, with a potential of 353 alleles.

The estimated allelic potential for the entire state of Hesse was 309, while the figure for NRW was 387. For the entire study area, the allelic potential was estimated to be 409.

The statistical analysis revealed that the population genetic parameters An, Na, He and Ar exhibited significant differences (P < 0.05) between the NRW and Hesse regions (Table 1 in S1 File Supplement, left). The effect of state was found to account for up to 22% of the total variance. No differences were observed for Ho and Fis, or Na(p). This suggests that within-state effects may be important in determining population genetic outcomes. Accordingly, the regions within each state were incorporated into the analysis. The initial comparison was between the Hessian and North Rhine-Westphalian AMUs within the topographical region of the Rothaargebirge (RHG).

The mean values obtained for the population genetic parameters An, Na(p), Na and Ar differed statistically significantly (P < 0.01) between the AMUs of the North Rhine-Westphalian and the Hessian side of the Rothaargebirge (Table 1 in S1 File Supplement, right), with higher mean values consistently observed in NRW. Furthermore, the values of He and Fis were found to be significantly higher in the region under consideration in the federal state of NRW than in the neighbouring state of Hesse(P < 0.05). With regard to the parameter Ho, no differences were observed between the federal states.

The AMUs of Eifel (EI), Moehnetal (MT), and the North Rhine-Westphalian part of the Rothaargebirge (RHG) exhibited markedly elevated numbers in both the overall (An) and the mean alleles (Na) when compared to the Hessian AMUs (Table 4). The corresponding values for Ar were also obtained, with the Möhnetal and Rothaargebirge in NRW exhibiting higher values than the Eifel. In similar manner, the expected heterozygosity (He) exhibited a comparable discrepancy. The observed heterozygosity (Ho) was found to be significantly higher in the Hessian part of the Rothaargebirge and exhibited a notable divergence from the Hessian Rheingau Taunus (RGT). Nevertheless, the highest Fis values were also observed in the Möhnetal, Rothaargebirge and Eifel regions of North Rhine-Westphalia. When extrapolated to the total population, the Eifel and the North Rhine-Westphalian part of the Rothaargebirge exhibited significantly higher values than all other areas. The lowest numbers were observed in the Vogelsberg (VB) and the northern part of the Ruhr (NR) area, which are directly comparable with the effective population size (Ne).

**Table 4:** Mean values of population genetic parameters by federal state and region.

		1 NRW Eifel	2 NRW NR	3 NRW Moehne	4 NRW RHG	5 Hesse RHG	6 Hesse LMR	7 Hesse VB	8 Hesse RGT
**An**	Mean±SD	155.25 ± 7.47	113 ± 5.28	154.6 ± 6.68	147.2 ± 6.68	117.75 ± 7.47	120.22 ± 4.98	113.33 ± 8.63	106.33 ± 8.63
	Pairwise P^*^	2,5-8	1,3-4	2,5-8	2,5-8	1,3,4	1,3,4	1,3,4	1,3,4
**Na**	Mean±SD	9.7 ± 0.47	7.06 ± 0.33	9.66 ± 0.42	9.18 ± 0.42	7.35 ± 0.47	7.51 ± 0.31	7.07 ± 0.54	6.63 ± 0.54
	Pairwise P	2,5-8	1,3,4	1,5-8	2,5-8	1,3,4	1,3,4	1,3,4	1,3,4
**He**	Mean±SD	0.74 ± 0.01	0.66 ± 0.01	0.73 ± 0.01	0.73 ± 0.01	0.7 ± 0.01	0.68 ± 0.01	0.68 ± 0.01	0.65 ± 0.01
	Pairwise P	2,5-8	1,3-5	1,5-8	2,6-8	1,2,3,8	1,3,4	1,3,4	1,3,4,5
**Ho**	Mean±SD	0.7 ± 0.02	0.66 ± 0.01	0.69 ± 0.01	0.7 ± 0.01	0.69 ± 0.02	0.67 ± 0.01	0.65 ± 0.02	0.64 ± 0.02
	Pairwise P	2,6-8	1,4	8	2,7,8	8		1,4	1,3-5
**Ar**	Mean±SD	6.34 ± 0.17	5.04 ± 0.12	6.35 ± 0.15	6.35 ± 0.15	5.54 ± 0.17	5.45 ± 0.11	5.41 ± 0.19	4.9 ± 0.19
	Pairwise P	2,5-8	1,3-6	2,5-8	2,5-8	1-4,8	1-4,8	1,3,4	1,3-6
**Fis**	Mean±SD	0.05 ± 0.02	0 ± 0.01	0.06 ± 0.01	0.05 ± 0.01	0.02 ± 0.02	0.03 ± 0.01	0.04 ± 0.02	0.02 ± 0.02
	Pairwise P	2	1,3,4	2,5,6	2	3	3		
**Na(P)**	Mean±SD	271.75 ± 19.91	151.63 ± 14.08	208.4 ± 17.8	248.2 ± 17.8	178.75 ± 19.91	186.33 ± 13.27	156.33 ± 22.98	183.33 ± 22.98
	Pairwise P	2,3,5-8	1,3,4	1,2	2,5-8	1,4	1,4	1,4	1,4
**Ne**	Mean±SD	555.25 ± 102.39	124.63 ± 72.4	190.6 ± 91.58	313.2 ± 91.58	238.5 ± 102.39	402.11 ± 68.26	135 ± 118.23	523.33 ± 118.23
	Pairwise P	2,3,5,7	1,6,8	1,8		1	2	1,8	2,3,7

NRW: North Rhine-Westphalia; RG: Rothaargebirge; VB: Vogelsberg; NR: Northern Ruhr area and other densely populated areas; RGT: Rheingau-Taunus area; LMR: Hessian low mountain ranges except RGT, VB and RG; for location of regions see Fig 1. Data are given as mean ± standard deviation.

* Pairwise P: numbers indicate to which other regions (as numbers) significant differences exist

### Effective population size

In the context of NRW, the effective population sizes estimated with NeEstimator were applied to ten AMUs that were clearly isolated and with an Nc < 1,000. In Hesse, only three AMUs comply with the standards for using the NeEstimator. In the case of the remaining AMUs, the demographically effective population size was employed as the mean value derived from the formulae of Wang et al. (2016) and Caballero (1994) due to proven connectivity. The smallest effective population size was observed in RK, with a value of 8.7. The largest demographically effective population sizes were calculated for the EZM (917), SP (802) and TAU (784) AMUs. The maximum estimated effective population size for the entire region was 11838. The highest net earnings ratio for individual AMUs in NRW was 515 for EZM (Table 5A). The remaining individual AMUs exhibited values below 500, with a total of nine AMUs falling below 100. A total of 6 AMUs achieved Ne values below 100 in Hesse (Table 5B). The Ne for the Eifel and Rothaargebirge in NRW exceeded 1,000, while the figure for the RGT in Hesse surpassed 500. The anticipated loss of heterozygosity per generation for individual AMUs exhibited a range of 0.04 to 5.75% in the context of NRW and a range of 0.1 to 1.94% in the context of Hesse.

**Table 5:** A. The effective population sizes of AMUs in NRW, as well as the regions and states according to NeEstimator, Wang et al. [57] and Caballero [58]. B. Effective population sizes of AMUs in Hesse, regions and states according to NeEstimator, Wang et al. [57] and Caballero [58].

A											
		NeEstimator Pcrit:					Ne demographic				
**State/Region/AMU**		**0.00**	**0.01**	**0.02**	**0.05**	**Taken**	**Wang**	**Caballero**	**Average** ^§^	**Ne/Nc**	**ELHG**
	NRW	211.4	128.9	105.4	76.5	211.4	5189.59	3109.64	**2836.88**	0.16	0.02%
**1**	EI	154.8	313.5	328.9	343.3	313.5	2226.21	1352.18	**1297.30**	0.16	0.04%
**1.1**	EHU	65.1	99.7	118.2	104.6	99.7	237.31	143.91	**160.31**	0.18	0.50%
**1.2**	ENP	131.2	224.9	259.5	246.2	224.9	672.26	402.72	**433.29**	0.19	0.12%
**1.3**	EZM	57.6	57.6	70.2	74.6	70.2	917.25	560.13	**515.86**	0.15	0.10%
**1.4**	EFW	87.8	116.3	136.7	107.0	116.3	395.15	241.76	**251.07**	0.17	0.20%
**2**	RK	8.7	8.7	8.7	13.4	**8.7**	43.80	25.76	26.09	0.18	5.75%
**3**	NR	43.8	64.3	59.8	64.1	64.3	369.47	223.93	**219.23**	0.16	0.23%
**3.1**	HX	28.5	53.4	61.4	52.6	**53.4**	95.31	57.77	68.83	0.20	0.94%
**3.2**	UF	38.8	64.9	108.3	113.8	64.9	274.16	166.16	**168.41**	0.17	0.30%
**4**	WH	89.1	99.7	116.3	91.9	**99.7**	98.65	58.92	85.76	0.25	0.50%
**5**	NS	17.7	17.7	17.7	6.8	**17.7**	19.38	11.59	16.22	0.24	2.82%
**6**	EB	22.4	22.4	19.3	24.7	**19.3**	48.10	28.06	31.82	0.20	2.59%
**7**	MT	87.0	82.7	80.8	75.5	82.7	305.08	183.98	**190.59**	0.18	0.26%
**7.1**	MOA	56.2	56.3	57.7	70.6	**56.3**	178.80	109.22	114.77	0.17	0.89%
**7.2**	MOB	105.1	93	87.3	104.2	**93.0**	120.72	71.56	95.09	0.23	0.54%
**8**	ETS	81.2	78.6	61.7	50.9	78.6	347.36	208.93	**211.63**	0.17	0.24%
**8.1**	EG	73.2	80.5	91.1	68.7	**80.5**	278.57	167.56	175.54	0.18	0.62%
**8.2**	SE	103.4	110.8	108.7	91.9	**110.8**	68.79	41.38	73.66	0.30	0.45%
**9**	MI	54.5	54.5	52.5	44.1	**52.5**	48.70	28.72	43.31	0.27	0.95%
**10**	RHG	283.5	269.3	230.0	198.7	269.3	2538.78	1502.25	**1436.78** ^*^	0.17	0.03%
**10.1**	SI	151.1	196.4	204.5	175.2	196.4	433.60	258.98	**296.33**	0.20	0.17%
**10.1.1**	SIW	89.7	89.7	107.7	78.8	107.7	171.12	102.21	**127.01**	0.22	0.46%
**10.1.2**	SIO	75.4	124.8	121.4	128.4	124.8	262.48	156.78	**181.35**	0.20	0.40%
**10.2**	WSS	156.0	168.2	151.2	92.5	168.2	635.00	371.18	**391.46**	0.19	0.13%
**10.2.1**	WGS	97.1	97.1	98.3	94.8	98.3	348.92	203.95	**217.06**	0.19	0.23%
**10.2.2**	BB	304.8	304.8	142.5	82.6	142.5	286.08	167.22	**198.60**	0.21	0.35%
**10.3**	WB	113.3	113.3	107.4	84.3	113.3	498.24	295.09	**302.21**	0.18	0.17%
**B**											
		**NeEstimator** **Pcrit:**					**Ne demographic**				
**State/Region/AMU**		**0.00**	**0.01**	**0.02**	**0.05**	**Taken**	**Wang**	**Caballero**	**Average** ^§^	**Ne/Nc**	**ELHC**
	HE	347.2	243.9	220.0	221.3	347.2	6655.01	3932.62	**3644.94**	0.16	0.01%
**10.4**	DB	39.3	37.2	42.3	43.5	37.2	340.97	201.57	**193.25**	0.17	0.26%
**10.5**	RGL	109.1	132.7	124.4	112.0	132.7	380.31	222.32	**245.11**	0.19	0.20%
**10.5.1**	RG	46.3	36.1	34.1	23.4	36.1	260.39	152.22	**149.57**	0.17	1.39%
**10.5.2**	LB	30.6	30.8	33	56.3	30.8	119.92	70.10	**73.61**	0.19	1.62%
**10.6**	BKW	212.5	183	165	119.5	183.0	233.29	137.99	**184.76**	0.24	0.27%
**11**	WW	31.6	31.5	29.6	24.1	**31.5**	17.24	9.73	19.49	0.33	1.59%
**12**	RW	70.4	159.9	168.4	148.9	**161.1**	204.67	120.23	162.00	0.24	0.31%

B											
**13**	NH	124.4	107.0	98.0	83.2	107.0	2026.28	1202.29	**1111.86**	0.16	0.04%
**13.1**	MKW	50	61.1	102.5	106.4	61.1	514.22	305.12	**293.48**	0.17	0.17%
**13.2**	RF	77.3	109.5	215.2	374.5	109.5	606.13	363.46	**359.69**	0.17	0.14%
**13.3**	KNU	76.8	81.6	80.4	81.4	81.6	404.37	239.47	**241.81**	0.18	0.21%
**13.4**	SW	174.3	215.8	170.3	262.3	215.8	493.58	289.77	**333.05**	0.20	0.15%
**14**	VB	73.1	83.2	77.6	80.0	83.2	404.54	241.86	**243.20**	0.17	0.21%
**14.1**	GF	55.6	49	42.3	44.4	49.0	107.17	65.43	**73.87**	0.18	1.02%
**14.2**	HV	44.5	44.5	53.9	65.6	53.9	238.80	140.34	**144.35**	0.18	0.93%
**14.3**	NV	47.3	39.5	40.1	38.1	39.5	58.74	34.68	**44.31**	0.22	1.27%
**15**	SP	368.5	183.7	206.4	205.6	183.7	801.65	477.43	**487.59**	0.18	0.10%
**16**	KF	46.8	44.2	40.1	38.3	**44.2**	47.23	26.93	39.45	0.25	1.13%
**17**	RGT	104.3	180.8	153.3	150.1	180.8	1591.08	940.43	**904.10**	0.17	0.06%
**17.1**	TAU	59	54.3	46.6	71.8	54.3	784.28	462.35	**433.65**	0.17	0.12%
**17.2**	PL	25	25	25.8	40	25.8	73.57	42.85	**47.41**	0.20	1.94%
**17.3**	HW	59.8	86.7	93.1	85.5	86.7	711.67	426.47	**408.28**	0.16	0.12%
**18**	OD	115.7	98.6	85.0	67.8	98.6	529.53	316.63	**314.92**	0.17	0.16%

Pcrit: critical value for allele frequency: 0.00: all allele singletons per omitted for Ne estimation; 0.05: alleles with frequencies less than 5% were omitted; ELHG: expected loss of heterozygosity per generation; effective population numbers were taken from NeEstimator (Do et al. [56]) or (§) as an average according to the formulas of Wang and Caballero [57,58]. The taken Ne is highlighted in bold numbers.

* : combined from AMUs in Hesse and NRW. Pcrit: critical value for allele frequency: 0.00: all allele singletons per omitted for Ne estimation; 0.05: alleles with frequencies less than 5% were omitted; ELHG: expected loss of heterozygosity per generation; effective population numbers were taken from NeEstimator (Do et al. [56]) or (§) as an average according to the formulas of Wang and Caballero [57,58]. The taken Ne is highlighted in bold numbers

### Allelic differentiation between AMUs

The highest degree of allelic differentiation, as indicated by Jost’s D values, was observed between RK and ENP (Jost’s D = 0.4997). In contrast, the lowest degree of differentiation was noted between SIO and WGS (Jost’s D = 0.0132) (Tables 2A–D in S1 File).

The lowest Fst value was also observed between SIO and WGS (Fst = 0.0084). The highest Fst value was found for the comparison between PL and RK (Fst = 0.252) (Tables 2A–D in S1 File).

### Genetic and geographic distance

**Fig 2:**
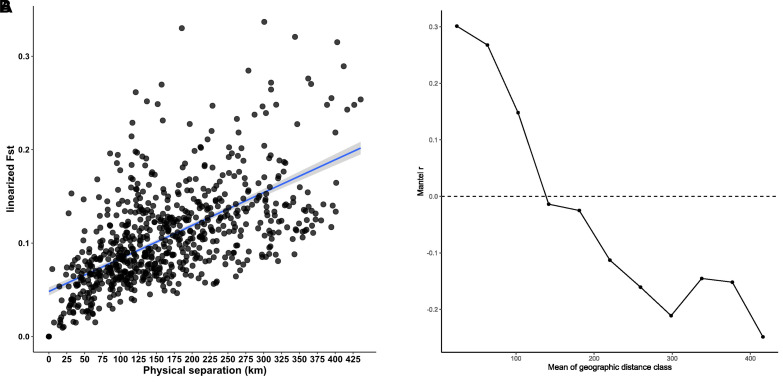
A. Variation of genetic distance (linearised Fst) between AMUs in Hesse and North Rhine-Westphalia 2020/2022, presented as a function of geographical distance. (Mantel test: r = 0.577, P* *= 0.0001). The blue lines and shaded area indicate the 95% confidence interval. B. The Mantel correlogram showing positive spatial autocorrelation up to a distance of about 122 km.

The results demonstrated a significant positive correlation between genetic distance and geographical distance, with an increase in geographical distance up to 122 km (p < 0.001). The Mantel test yielded a correlation coefficient of 0.577. This indicates that 33.2% of the observed genetic distance can be attributed to the influence of IBD (isolation by distance) (see Figs 2 A and 2B).

### Genetic structure analysis by clustering algorithms

**Fig 3:**
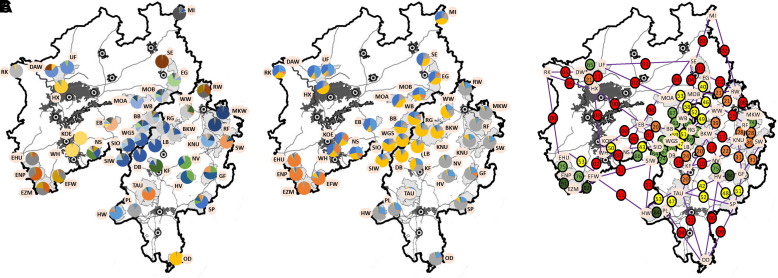
A. Distribution of Clusters according to STRUCTURE. DAW = 7 samples; KOE = 4 samples. Maps were constructed in QGIS, Version 3.28.3. B. Distribution of Clusters according to TESS. DAW = 7 samples; KOE = 4 samples. Maps were constructed in QGIS, Version 3.28.3. C. Connectivity between neighbouring AMUs based on the agreement of joint cluster prevalence (including STRUCTURE, DAPC, BAPS and TESS). Numbers in circles represent the connectivity. Dark green colour: connectivity between two neighbouring AMUs of at least 80%. Light green colour: connectivity between two neighbouring AMUs 60 to 79%; yellow colour: connectivity between two neighbouring AMUs 40 to 59%; orange colour: connectivity between two neighbouring AMUs 20 to 39%; red colour: connectivity between two neighbouring AMUs lower than 20%. Maps were constructed in QGIS, Version 3.28.3.

The hierarchical STRUCTURE analysis classified the 2490 individuals into four clusters at the first level of classification. The clusters were named as follows: Eifel, Rothaargebirge, Centre, East to North NRW (including Rothaargebirge), Northwest NRW and Hesse with the exception of the AMUs of the Rothaargebirge. The Dirichlet parameter indicated a restricted degree of admixture. At the second level of analysis, the four initial clusters were further subdivided into six, three, four, and two clusters, respectively. The informativeness r of the LOCPRIOR was consistently less than one, indicating that the information about the sampling location was useful for assigning individuals to clusters. Ultimately, 33 clusters were identified (see Fig 3A). The distribution of clusters within each red deer area permitted a visual assignment of genetic differentiation between areas, as well as an initial categorisation of the genetic diversity present. The highest level of genetic variability was attributed to the Rothaargebirge region, which exhibited the greatest number of clusters per AMU. AMUs with a limited number of dominant clusters were idendified in MI, RK, WH/KOE, SE, MKW/RF, PL and OD, in a northwest-southwards progression. The number of alleles differed between clusters. The number of specific alleles within a given cluster ranged from 75 (cluster 24) to over 170 (clusters 23, 25, 27). The Eifel region provides an illustrative example of this phenomenon, exhibiting a high level of allelic diversity within a relatively small number of clusters.

In the DAPC clustering analysis (K = 2–50), the minimum BIC values were observed with K = 26. Consequently, a DAPC analysis was conducted with 26 clusters and 211 retained principal components, which collectively explained 100% of the genetic variance. The DAPC clusters were found to be in close agreement with the STRUCTURE clusters (see Fig 1 in S1 File). A significant discrepancy was observed in the EB region, which was assigned to a single cluster in the DAPC analysis. It can be assumed that a significant involvement of a cluster in an area with a prevalence of 5% or more will result in the exclusive occurrence of clusters 5, 18, 19, 20, 21, 22, 24, 25 and 26. This is evidenced by the fact that these clusters occurred exclusively in EB, OD, SE, UF, WH, MKW, NR, MI and RK, respectively. This indicates the isolation of these areas. Conversely, clusters 1, 4, 6, 10 and 17 were present in at least 10 AMUs and in AMUs BB, MOB, RG, SIW and WB, with a prevalence of > 5% in animals from at least six clusters.

BAPS was employed to identify 31 clusters for the purpose of characterising genetic variability. This resulted in a greater level of differentiation between AMUs than that achieved by STRUCTURE and DAPC, and it could be used to gain further insight into regions with poorly differentiated AMUs (Fig 2 in S1 File).

TESS provided a highly conservative representation (Fig 3B). The genetic characteristics of the populations were divided into a mere five clusters, with minimal differentiation observed between AMUs. However, this enabled the observation of relationships over longer distances, thereby facilitating the examination of a longer time horizon. This revealed the existence of four areas, namely the Eifel in the south-west, Hesse in the south-east, the Rothaargebirge in the centre, and the north-west of NRW, each with fluid transitions, and a fifth cluster without a clear regional assignment. These correlations were not identified by any of the other selected algorithms.

### Genetic connectivity between populations

**Fig 4:**
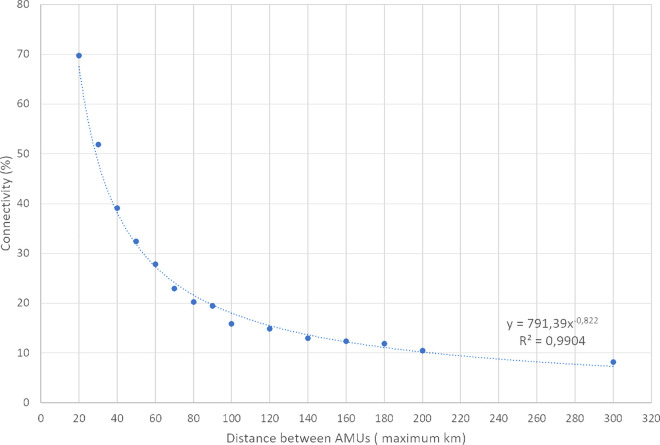
Mean connectivity between AMUs depending on the geographical distance between AMUs. E.G.: dot at 80 = distance >60 and <81 km. Trend line with formula and coefficient of determination (R^2^).

To facilitate the quantification of the connectivity between neighbouring AMUs, the percentage agreement of cluster prevalence from STRUCTURE, DAPC, BAPS and TESS was employed (Fig 3C). Genetic connectivity and geographic distance exhibited a strong correlation, following a power function with a negative odd exponent (see Fig 4). The x-axis of Fig 4 depicts the maximum distance between AMUs in 10 km increments up to 100, 20 km increments up to 200, and all AMU pairs with a distance exceeding 200 km. The point at 60 on the x-axis represents the connectivity of all AMU pairs with a geographical distance between 41 km and 60 km. The initially decrease in connectivity is steep, reaching a distance of 40 km and a connectivity of 40%. Subsequently, from approximately 80 km and a connectivity of 20%, the decrease in connectivity is relatively gradual (Fig 4).

**Fig 5:**
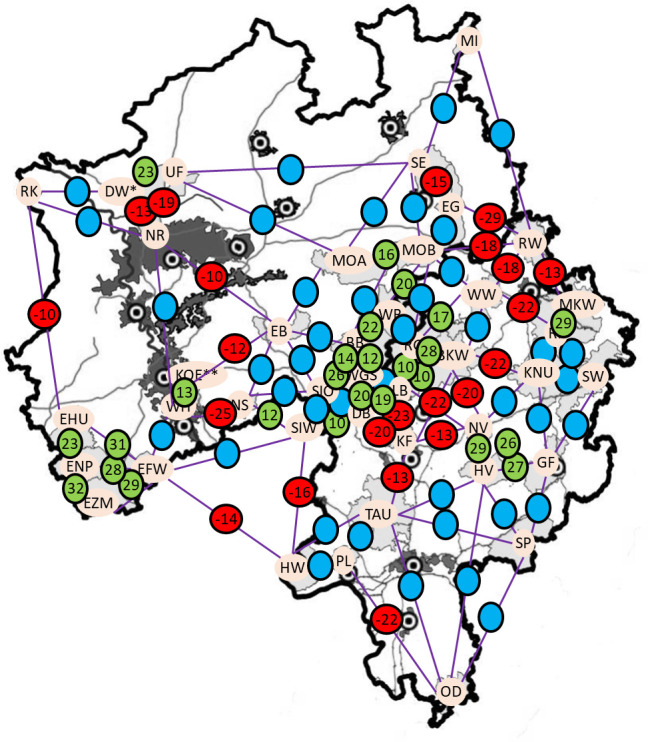
Deviation from target connectivity according to Fig 4. The figures displayed in the circles illustrate the discrepancy between the actual connectivity between two neighbouring AMUs and the connectivity calculated on the basis of geographical distance. Green circles: actual connectivity was at least 10% higher than the calculated connectivity; red circles: actual connectivity was at least 10% lower; blue circles: actual and calculated connectivity differed by less than 10%. DAW = 9 samples; KOE = 4 samples. Maps were constructed in QGIS, Version 3.28.3 [71].

**Fig 6:**
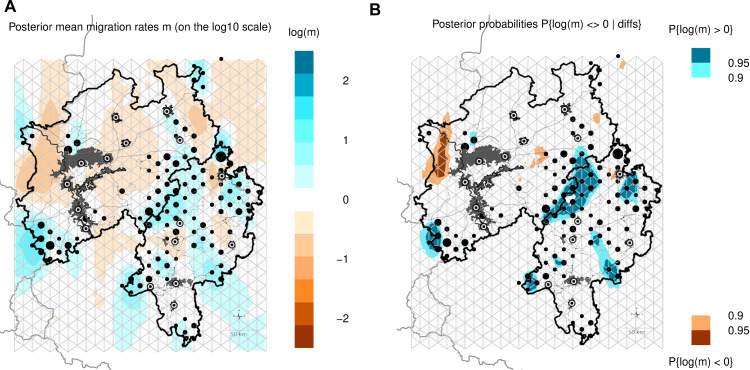
Estimated effective migration surface (EEMS) according to Petkova et al. [60]. The triangles represent the 660 pre-defined demes. Black spots show sampling locations. The larger the spot, the greater the number of samples. Green to blue areas show over-average migration in a log 10 manner. Fig 6A shows the posterior mean migration rates m, with orange and red areas indicating under-average migration. Fig 6B shows the statistical significance (p) of the migration rates in Fig 6A. The additional outlines of the study area were constructed in QGIS, version 3.28.3.

The calculated connectivity based on geographical distance (Fig 4) did not always align with the actual connectivity between AMUs. The corresponding deviations are illustrated in Fig 5. In certain regions, particularly in the Eifel (EI) and Rothaargebirge (RHG) areas, but also in the VB region, between RF and MKW, the Möhnetal (MT) region, between NS and SIW, the KOE and WH, and the DAW and UF, the connectivity between neighbouring areas was at least 10% higher than would be expected from the geographical distance alone (green dots, Fig 5). Conversely, lower values were observed along the A5, in the vicinity of the RW, the KF, between the EG and the SE, and from the NR area to the south-east (Fig 5, red dots). The remaining area comparisons were largely in accordance with expectations based on their geographical distances (Fig 5, blue dots). Fig 5 thus represents the deviation between neighbouring areas beyond their geographical distance, thereby enabling inferences to be drawn about the relative barrier function of each region. This further illustrates the enhanced connectivity within the Eifel and Rothaargebirge regions, as well as in the VB area, between MKW and RF, MOA and MOB, WH and KOE as well as UF and DAW. These findings are supported by the EEMS. However, the low-differentiated region of the Eifel has shifted westwards, and the large urban area between NR in the north-west and the Eifel in the south-west shows average migration. Furthermore, the RHG appears to be connected to the VB region in Fig 6A. However, this connection lacks statistical significance (see Fig 6B).

The connectivity between AMUs with a maximum geographical distance of 40 km reached 46% in Hesse, which is only 89% of the North Rhine-Westphalian values (51%) (Fig 3 in S1 File). This discrepancy was statistically significant. It tended to persist when comparing the Hessian side of the Rothaargebirge with the North Rhine-Westphalian side. The superiority of the AMUs in North Rhine-Westphalia is particularly attributable to the maximum connectivity observed in the Eifel region, which reached 72%. For the smaller, isolated AMUs (LMR in Hesse and NR in NRW), the mean connectivity between sites was below 40%. The mean connectivity of the individual AMUs is illustrated in Fig 4 in S1 File. Areas of the Eifel region exhibited connectivity levels exceeding 70%, whereas the northern Ruhr (NR) and Nutscheid (NS) areas demonstrated connectivity levels of approximately 20% even at a maximum distance of 40 km. The SE, EG, KOE and WH in NRW and a number of AMUs in Hesse, which are considered to be isolated, also exhibited connectivity levels that were approximately half those observed in the Eifel area.

**Fig 7:**
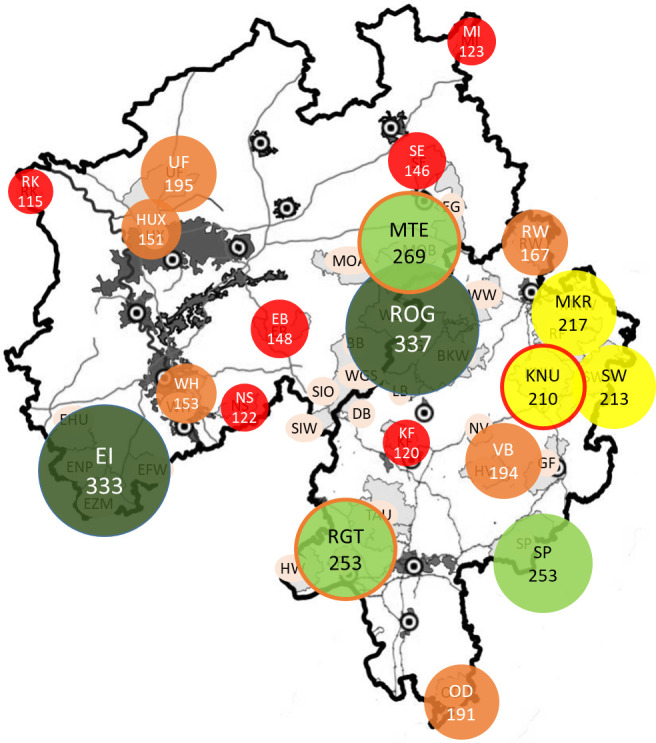
Number of alleles based on the current population size (Na(p)). Maps were constructed in QGIS, Version 3.28.3. Colour coding of populations according to allele potential with red (0-150), orange (151-200), yellow (201-250), light green (251-300) and dark green (> 300).

**Fig 8:**
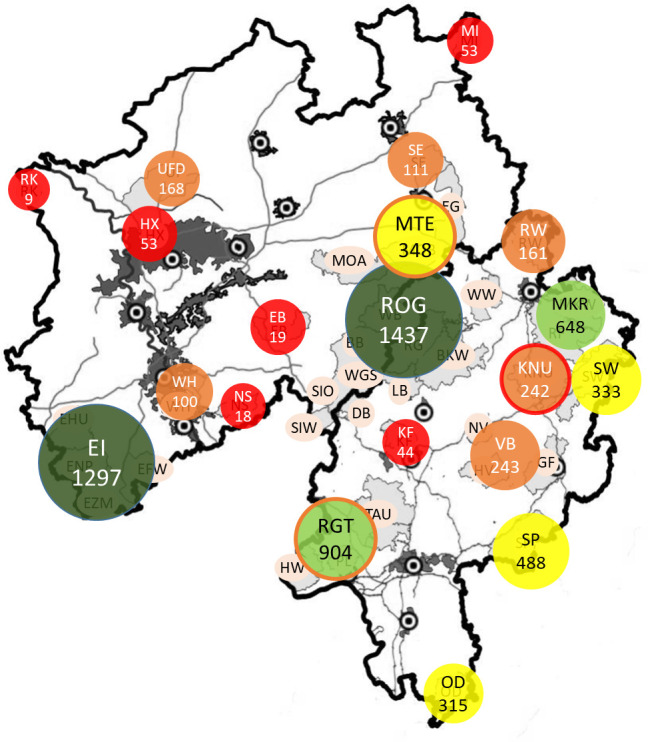
Effective population size (Ne) estimated for isolated AMUs and connected regions. Maps were constructed in QGIS, Version 3.28.3. Colour coding of populations according to effective population size with red (0-100), orange (101-250), yellow (251-500), light green (501-1000) and dark green (> 1000).

In consideration of the connectivity between AMUs, a distinction was made between isolated areas and those that were grouped into regions. For these regions, the number of alleles that should be present (Na(p)) was calculated based on the actual estimated number of animals (Fig 7). The lowest number of alleles was observed in the most isolated areas, namely RK (n = 115), KF (n = 120), NS (n = 122), MI (n = 123) and EB (n = 148). The Eifel (EI) and the Rothaargebirge (RHG) exhibited the highest number of alleles, with 333 and 337, respectively. The effective population sizes are illustrated in Fig 8.

Of the 40 AMUs included in the study, 14 were situated within two expansive regions: the topographical Rothaargebirge (RHG) (AMUs BB, BKW, DB, LB, RG, SIW, SIO, WW, WB, WGS) and the Eifel (EHU, ENP, EZM, EFW). The AMUs within each region are well connected to each other and both regions have reached effective population sizes of over 1,000. The Rheingau-Taunus area [RGT] (comprising the TAU, PL, HW) in Hesse also exhibited an effective population size approaching 1,000.

Two additional AMUs (5%), MKW and RF in Waldhesse (NH), exhibited values between 500 and 1,000. Both AMUs exhibited a high degree of genetic linkage, yet displayed pronounced differentiation for the remaining AMUs. A further 25% of AMUs with connectivity to neighbouring areas exhibited effective population sizes between 100 and 500. These were observed as a single area (SP, SW) or as a region (Möhnetal (MOA and MOB, ETS; VB and UF with DAW), with high connectivity within the region. A further 27.5% were found to be highly isolated. These included two AMUs with Ne > 100 (RW and KNU) and nine AMUs with Ne ≤ 100 (RK, HX, EB, NS, KF, MI, WH with KOE, and SE).

**Fig 9:**
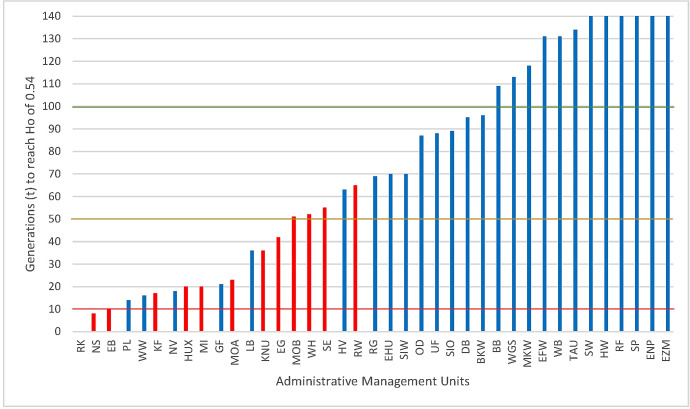
Classification of threat of populations according to Willoughby et al. [72] Red bars: threatened populations because of isolation; blue bars: unthreatened populations, because they are still connected with neighbours. According to Willoughby et al. [72] populations with values ≤ 10 (below red line) are critical endangered, those with values > 10 and ≤ 50 (below orange line) are endangered and those >50 and ≤ 100 (below green line) are vulnerable. The Y-axis shows the number of generations until a Ho of 0.54 is reached, corresponding to the 25% percentile of heterozygosity of the studies evaluated by Willoughby et al. [72].

For the classification of the threat status of the investigated red deer populations, we have applied the criteria of Willoughby et al. [72]. The red columns represent isolated AMUs for which the calculated criteria are directly applicable (Fig 9). The blue columns show the corresponding criteria for the still connected AMUs. They would only apply in case of their future isolation. The thresholds indicate 3 critically endangered populations (below the red line, ≤ 10), 6 endangered populations (below the orange line, ≤ 50) and 4 vulnerable populations (below the green line, ≤ 100). All populations above the green line can be considered non-threatened according to the criteria. Critically endangered populations are thought to reach homozygosity in 25% of their loci within just up to 10 generations.

## Discussion

The present study demonstrated that 35% of the AMUs examined exhibited high levels of isolation. The degree of isolation was quantified as reduced gene flow, expressed as the probability that individuals from two AMUs belong to the same cluster [28]. Isolation was deemed to have occurred when the connectivity between AMUs was less than 40%, and high isolation was considered to have occurred when the connectivity was approximately 20% or less [28]. The AMUs in NRW most affected by this phenomenon were RK, MI, SE, DW, UF, HX, WH, KOE and EB. In Hesse, the areas most affected were KF, OD, KNU, RW and SW. From a scientific perspective, it is hypothesised that restricted gene flow increases genetic drift and, consequently, the risk of loss of genetic diversity [8,12,19,29,73–77]. Indeed, a reduction in genetic diversity was observed with regard to various aspects of allelic diversity and effective population size, with a notable impact on the small isolated areas of RK, MI, EB, NS, SE, KF and HX. The number of alleles in these AMUs was found to be 16% lower than the expected value, with a further 40% reduction in the number of alleles based on the percentage of sampled individuals in the population (Na(p)). The unfavourable AMUs exhibited a 16% higher prevalence of animals with homozygosity levels exceeding 50% compared to the other AMUs. It is imperative that such small, isolated AMUs be regarded as particularly vulnerable [11,12,16,78]. This is because it is in small, isolated populations that rare gene variants can be lost, often with the loss of individual animals [79,80]. In the case of the AMU KF, the decline in genetic diversity from the 1980s to the 2010s has been demonstrated to be as high as 15% [36]. The reduction in allelic diversity and overall genetic diversity, as a consequence of allelic losses and genetic depletion, respectively, results in a decline in heterozygosity [12,81,82]. The current study provides evidence of this phenomenon, with the most severely affected areas exhibiting the lowest expected and observed heterozygosity, and thus the highest levels of homozygosity. In the long term, such a reduction in genetic variability could result in increasing levels of inbreeding [16,81,83] and inbreeding depression [13–15]. The issue of inbreeding depression, manifested as regular occurrences of malformations, is particularly evident in an area of Schleswig-Holstein, Germany, where red deer has been placed on the Watch List of the IUCN Red List of Threatened Species [13]. Furthermore, malformations associated with higher degrees of homozygosity have also been identified in the current study area in Hesse [28]

In contrast, 47.5% of the AMUs could be grouped into regions with total effective population sizes above 500 (including 42.5% with Ne > 1,000) due to their preserved connectivity. The remaining AMUs (22.5%) exhibited partial connectivity within limited regions, such as the Vogelsberg area (NV, HV, GF).

However, they were largely isolated from neighbouring regions with Ne above 100 but below 500. This classification is in accordance with the results previously published by Reiner et al. [28]. However, the connectivity and degree of isolation of the AMUs in the border area between Hesse and North Rhine-Westphalia, specifically the topographical region of the Rothaargebirge with the AMUs DB, LB, RG and BKW, could not be fully assessed in the earlier study due to limitations in the scope of the investigation. Only through a combined consideration of both states could the existing connectivity between them be accurately validated. It may be reasonably assumed that similar effects will be observed in other AMUs that border Bavaria, Lower Saxony or Rhineland-Palatinate, as well as in other studies.

The AMUs exhibiting the highest genetic diversity and genetic connectivity were situated in densely forested low mountain ranges (Eifel (EHU, ENP, EZM, EFW), Rothaargebirge (WB, BB, WGS, SIO, SIW, DB, LB, RG, BKW), Northeast Hesse (MKW, RF)) with minimal anthropogenic pressure. The AMUs exhibiting the lowest diversity and connectivity were among the smallest areas with the lowest demographic red deer population size. They were situated at a greater distance from neighbouring AMUs and in closer proximity to areas characterised by high human population density and extensive infrastructure. A substantial body of evidence attests to the threat to *Cervus elaphus* in various European locations due to isolation caused by a range of barriers [10,18,29,75,84–86]. The primary causes are the presence of dense settlements [7] and transport infrastructure [8,9]. [7,8,74,75,87,88]However, decresed connectivity was also observed in regions devoid of motorways or dense human settlements, particularly in the northern area beyond the Krofdorfer Forst. In this region, deer migration appears to be reduced by hunting in the ‘red deer-free areas’ between KF, DB and LB [28,89], a phenomenon that has also been observed in other regions [29]. In Germany, legislation indirectly restricts migration, as evidenced by the German Hunting Act in 2015. The designed red deer areas as administrative units for the management of game was initiated in the 1950s in response to the prevalence of damage caused by game at that time. A shooting plan was devised for each AMU, with the primary objective being to control the level of bark stripping in beech and spruce. The AMUs are situated within wooded areas where red deer populations have historically been present. These areas essentially represent the remaining historic summer habitats of red deer, while the former winter habitats in the floodplains have either disappeared or become inaccessible. In the ‘red deer-free areas’ between AMUs, the introduction of deer populations should be prevented in order to safeguard the vegetation from damage. The hunting of these animals impedes the migration of red deer between AMUs [88]. [7,17,20,35,82,83,90–95]

The differentiation between AMUs could be inferred from the basic population genetic data, but the application of Bayesian methods and their quantification significantly enhanced this process. The results demonstrate that the Bayesian methods STRUCTURE, BAPS and TESS, and DAPC as a multivariate method based on classical statistics, were able to provide significant additional insights into the distribution of Fst values, with R^2^ values of 52.6%, 26%, 16.8% and 9.6%, respectively. The highest level of agreement was observed between STRUCTURE and DAPC methods, with a correlation coefficient of (r = 0.82). In contrast, BAPS achieved a more refinded differentiation of even the less differentiated AMUs, which could prove useful for further differentiation within regions of high connectivity. Conversely, TESS permitted assignment over greater spatial distances due to low differentiation between AMUs, a feature not achieved by the other algorithms. This effect may be interpreted as an additional insight into the historical development of the present-day AMUs. It provides valuable insight into the genetic relationship between more distant areas. TESS demonstrated a high level of agreement between the AMUs of North Rhine-Westphalia, with the exception of the Eifel region [EI]. In contrast, the Hessian AMUs located both within and outside the Rothaargebirge [RHG] exhibited a notable degree of differentiation, despite the relatively low anthropogenic influence compared to the highly populated NRW region. It should be beneficial for future studies to address this question.

Zachos et al. [10] employed the NeEstimator, a linkage disequilibrium approach, to calculate effective population size, requiring a minimum of 25 samples analysed by at least 10 microsatellites. The authors themselves asserted the reliability of this method up to a Ne value of 200. As previously stated by Waples and England [71], it is essential to ascertain the migratory patterns between and within the populations of interest. Accordingly, in the present study, the NeEstimator values were applied exclusively to small and genuinely isolated AMUs. For larger AMUs (Nc > 1000) and AMUs that could be merged into regions based on calculated connectivity, a synthenic Ne was calculated from demographically estimated animal numbers following the methods of Wang et al. [57] and Caballero [58]. The rationale for this approach was that higher numbers of animals and greater habitat structuring are more likely to result in the formation of subpopulations, which can lead to deviations in linkage disequilibrium and thus misjudgments by the Ne estimator [10,71]. It can therefore be surmised that the results presented here are likely to be an overestimation in comparison to those reported by Zachos et al. [10] for Europe. Based on the NeEstimator evaluation alone, none of the AMUs examined in Hesse and NRW reached the long-term goal of 1,000, and not even 500.

A significant correlation between genetic and geographical distance was identified through regression analysis and subsequently validated by Mantel’s test. It was thus feasible to derive and identify target values for genetic distances between neighbouring areas, corrected for their geographical distance, thereby obtaining indications of barrier functions in the corresponding regions from the actual connectivity. In the case of Hesse, a broad barrier band was confirmed to extend from Kassel along the A7, A5 and A45 motorways. In the case of North Rhine-Westphalia, the most impenetrable regions were distributed in a more diffuse manner. It was found that the geographical distance between the areas in NRW (34,000 km², 20 AMUs) contributed more to the differentiation than in Hesse (21,000 km², 19 AMUs). In order to calculate and assess connectivity, only AMUs with a distance of 40 km or less were included in the comparison. With an average distance of 28 km, this was found to correspond well to the assumed movement radius of red deer [23]. It can be reasonably assumed that distances beyond this are less associated with direct movement activity and are influenced by a variety of non-specific factors.

The relationship between geographical distance and genetic differentiation can be explored using the EEMS programme to show deviations in gene flow through corridors or barriers, compared with deviations caused solely by isolation by distance [60]. Indeed, the connectivity analyses show good agreement with the EEMS results. These results clearly highlight the differentiation of the AMUs RK, MI and RW, as well as the barrier effect of a central band running from north to south through North Rhine-Westphalia, which separates NR from the rest of the study area. According to the EEMS, the demarcation of the OD in the south from the rest of the study region appears to be a mixture of IBD and anthropogenic infrastructure. The known barrier effect of the fenced motorways A45 and A5, to the west and south-east of the KF, is also clearly identified. Significant genetic exchange within the Eifel and the RHG, resulting from large contiguous habitats with little anthropogenic infrastructure, becomes clearly apparent, especially when considering the p values. EEMS tends to indicate average migration rates in the absence of a data basis, as can be seen in the heavily urbanised area between North Rhine-Westphalia’s northwest and the Eifel in the southwest, where red deer populations cannot exist. Regions without a solid data basis, also outside the study area, cannot therefore be assessed [60]. It should also be noted that the average migration according to EEMS refers to a relatively high degree of differentiation within the study region as a whole.

The challenge of comparing AMUs across vast geographical distances is exemplified by the work of Westekemper [22]. In this instance, only one AMU (DAW) in the far north-west was included for the entirety of North Rhine-Westphalia, while only five AMUs were included for Hesse (KF, OD, SP, TAU, RF). No evidence of connectivity was identified between the Hessian and North Rhine-Westphalian regions. However, the entire Rothaargebirge [RHG], which proved to be the most interesting and diverse area in the present study, was not included in the study by Westekemper [22]. This demonstrates that sufficient resolution can only be attained and valid assertions can only be made if all extant AMUs within the study area are incorporated. Concurrently, a multitude of assertions can be made regarding specific AMUs and potential avenues for enhancing future management, which were not feasible to present in the present study. The requisite number of samples was obtained for each area, with a few exceptions [35]. One exception was the comparison of the WH with the KOE. In combination, these two neighbouring areas were entirely isolated from all other AMUs by human infrastructure and settlements. But the two areas themselves are connected by a wildlife bridge spanning the A3 motorway, a railway line and a main road. Despite the limited sample size from KOE (n = 4), it was possible to infer connectivity between the two AMUs based on the genetic characteristics of these four individuals. Their genetic profiles did not align with other neighbouring AMUs, but instead exhibited a high degree of similarity to the WH. However, due to the limited sample size, no further population genetic parameters were calculated for KOE.

The notable genetic divergence between red deer populations across Europe, as outlined by Zachos et al. [10], can be substantiated within the context of the study region. This illustrates that the beneficial effects of extensive habitats and relatively high animal numbers are, to some extent, counterbalanced by the isolation of populations [11,96]. The resulting alarmingly low effective population sizes and inbreeding [10] have also been confirmed for the study area. Nevertheless, red deer populations with high genetic diversity and effective genetic exchange can still be found in less densely populated areas with less infrastructure. The reconnection of AMUs could prove an effective means of enhancing the status of small, isolated AMUs and ensuring the long-term survival of these valuable populations as part of the wider red deer species.

Populations in isolated and fragmented habitats lose genetic variability very quickly and are usually of conservation concern because they are at greater risk of local extinction [97], as what was once a continuous population becomes a series of smaller habitat patches [12]. This is clearly illustrated by the populations organised into AMUs in the present study, which are largely isolated from each other. Because the current populations are small and gene flow between them is low, genetic drift and inbreeding lead to loss of genetic variability, which can result in reduced survival and reproductive capacity of individuals [97]. However, such populations with low genetic diversity are overlooked by the current IUCN methodology, except in cases of drastically reduced population size [72,97]. The problem lies in the fact that heterozygosity and allelic richness, the main criteria in conservation genetics, are not adequately reflected in the previous IUCN criteria of ‘extent of range’, ‘number of mature individuals’ and ‘reduction in population size’ [72,97]. In an analysis of over 5,000 articles covering almost 18,000 microsatellite loci in 5,900 populations of over 1,300 species, they developed a new criterion capable of identifying populations at risk due to low genetic diversity, using heterozygosity and effective population size to estimate the number of generations required for heterozygosity to fall below 0.54, representing the 25% quantile of the studies screened by Willoughby et al. [72]. Using this new criterion, 7.5%, 15% and 10% of the isolated populations in the present study met the criteria for critically endangered, endangered and vulnerable populations, respectively. Further populations would be added if their isolation is not addressed in time. As each population may harbour unique genetic adaptations essential for the survival of the species in future climates [98], our results indicate the need for reconnection and corridors between AMUs.The significant discrepancies between AMUs demonstrate the necessity for a nuanced approach to the management of red deer, one that does not rely on generalised assumptions at the federal state level. It is therefore necessary to consider local conditions, which are also associated with considerable differences in genetic diversity and connectivity within countries. The majority of the AMUs analysed exhibited low genetic diversity and connectivity in comparison with the better AMUs. These were associated with a reduction in effective population size and an increase in homozygosity. The smaller and more isolated AMUs have a markedly disruptive effect on genetic exchange throughout the study area. In order to address this issue, it is necessary to implement adaptations to the management of red deer in the region.

## Conclusions

In conclusion, this study provides relevant information for the conservation of a native deer species that needs to be considered in future conservation strategies. It highlights the importance of using genetic data to assess extinction risk, rather than just population size. Finally, it shows that microsatellites remain a valuable and useful tool for assessing threatened species.

## Supporting information

S1 File Supplemental tables and figures.
(DOCX)
