# Supplementary material for: High-resolution analysis of red deer (Cervus elaphus) management units in a Central European region of high human population density reveals severe effects on genetic diversity and differentiation
Source: PLoS One. 2025 Jun 27;20(6):e0327427. doi: 10.1371/journal.pone.0327427 (PMC12204628; doi:10.1371/journal.pone.0327427)
Supplement: S1 File — Supplemental tables and figures. (DOCX) [file pone.0327427.s001.docx]

**High-resolution analysis of red deer (*Cervus elaphus*) management units in a Central European region of high human population density reveals severe effects on genetic diversity and differentiation.**

Julian Laumeier, Corinna Klein, Hermann Willems, Gerald Reiner

Supplementary Tables and Figures

**Table 1:** **Differences in population genetic parameters (Mean ± SD) between North Rhine Westphalia (NRW) and Hesse (all AMUs included vs. only AMUs located in the region of the Rothaargebirge).**

|  | **All AMUs included** | | | | **Only AMUs located in the Rothaargebirge** | | | |
| --- | --- | --- | --- | --- | --- | --- | --- | --- |
|  | **NRW (n=21)=)** | **Hesse (n=19)=)** | **P** | **R2** | **NRW (n=5 )** | **Hesse (n=4)** | **P** | **R2** |
| **An** | 135.2 ± 24.4 | 117.2 ± 11.7 | 0.005 | 18.8 | 147.2 ± 10.4 | 117.8 ± 9.6 | 0.003 | 73.1 |
| **Na** | 8.4 ± 1.5 | 7.3 ± 0.7 | 0.005 | 18.9 | 9.2 ± 0.64 | 7.4 ± 0.58 | 0.003 | 73.7 |
| **He** | 0.71 ± 0.04 | 0.68 ± 0.02 | 0.031 | 11.3 | 0.73 ± 0.017 | 0.70 ± 0 | 0.011 | 66.7 |
| **Ho** | 0.68 ± 0.04 | 0.67 ± 0.03 | 0.081 | 6.8 | 0.70 ± 0.017 | 0.69 ± 0.01 | n.s. | 0.0 |
| **Ar** | 5.9 ± 0.7 | 5.4 ± 0.4 | 0.014 | 14.8 | 6.4± 0.22 | 5.54 ± 0.30 | 0.002 | 76.1 |
| **Fis** | 0.032 ± 0.039 | 0.025 ± 0.022 | 0.535 | 0.0 | 0.045 ± 0.017 | 0.018 ± 0.012 | 0.001 | 66.7 |
| **Na(p)** | 206.7 ± 62.9 | 180.2 ± 38.2 | 0.116 | 6.4 | 248.2 ± 24.3 | 178.8 ± 24.3 | 0.004 | 72.2 |

An: Absolute number of alleles per AMU; Na: average number of alleles per marker; He: expected heterozygosity; Ho: observed heterozygosity; Fis: inbreeding coefficient according to Wright; Ar: allelic richness; Na(p): number of alleles based on estimated total population size; Ne: effective population size; P: significance; R^2^: coefficient of determination

**Table 2A Table:** **Fst values (below the diagonal) and Jost's D values (above the diagonal) for the Hessian AMUs.**

|  | **BKW** | **DB** | **GF** | **HV** | **HW** | **KF** | **KNU** | **LB** | **MKW** | **NV** | **OD** | **PL** | **RF** | **RG** | **RW** | **SP** | **SW** | **TAU** | **WW** |
| --- | --- | --- | --- | --- | --- | --- | --- | --- | --- | --- | --- | --- | --- | --- | --- | --- | --- | --- | --- |
| **BKW** |  | 0.072 | 0.136 | 0.13 | 0.15 | 0.129 | 0.109 | 0.065 | 0.18 | 0.128 | 0.215 | 0.176 | 0.147 | 0.027 | 0.088 | 0.12 | 0.156 | 0.116 | 0.068 |
| **DB** | 0.039 |  | 0.13 | 0.154 | 0.16 | 0.084 | 0.174 | 0.054 | 0.178 | 0.138 | 0.218 | 0.166 | 0.153 | 0.041 | 0.153 | 0.127 | 0.123 | 0.11 | 0.098 |
| **GF** | 0.079 | 0.077 |  | 0.055 | 0.125 | 0.129 | 0.171 | 0.153 | 0.169 | 0.065 | 0.226 | 0.107 | 0.129 | 0.1 | 0.118 | 0.085 | 0.089 | 0.095 | 0.172 |
| **HV** | 0.068 | 0.08 | 0.035 |  | 0.147 | 0.153 | 0.205 | 0.152 | 0.168 | 0.036 | 0.208 | 0.159 | 0.121 | 0.139 | 0.103 | 0.106 | 0.118 | 0.113 | 0.132 |
| **HW** | 0.082 | 0.085 | 0.066 | 0.084 |  | 0.128 | 0.108 | 0.151 | 0.153 | 0.13 | 0.163 | 0.046 | 0.114 | 0.115 | 0.112 | 0.145 | 0.092 | 0.064 | 0.148 |
| **KF** | 0.075 | 0.057 | 0.061 | 0.083 | 0.077 |  | 0.167 | 0.124 | 0.159 | 0.157 | 0.24 | 0.155 | 0.128 | 0.094 | 0.162 | 0.123 | 0.119 | 0.149 | 0.094 |
| **KNU** | 0.058 | 0.079 | 0.076 | 0.079 | 0.061 | 0.076 |  | 0.185 | 0.187 | 0.167 | 0.214 | 0.166 | 0.13 | 0.134 | 0.119 | 0.173 | 0.127 | 0.172 | 0.12 |
| **LB** | 0.033 | 0.026 | 0.082 | 0.077 | 0.082 | 0.069 | 0.08 |  | 0.183 | 0.104 | 0.217 | 0.169 | 0.151 | 0.051 | 0.153 | 0.152 | 0.158 | 0.118 | 0.096 |
| **MKW** | 0.088 | 0.086 | 0.092 | 0.096 | 0.078 | 0.088 | 0.093 | 0.086 |  | 0.163 | 0.17 | 0.173 | 0.042 | 0.189 | 0.137 | 0.136 | 0.106 | 0.134 | 0.158 |
| **NV** | 0.074 | 0.076 | 0.04 | 0.02 | 0.072 | 0.074 | 0.075 | 0.063 | 0.083 |  | 0.183 | 0.146 | 0.146 | 0.115 | 0.121 | 0.124 | 0.145 | 0.117 | 0.134 |
| **OD** | 0.101 | 0.107 | 0.112 | 0.107 | 0.102 | 0.12 | 0.103 | 0.113 | 0.096 | 0.097 |  | 0.234 | 0.183 | 0.191 | 0.149 | 0.143 | 0.185 | 0.169 | 0.202 |
| **PL** | 0.096 | 0.093 | 0.08 | 0.098 | 0.038 | 0.087 | 0.083 | 0.093 | 0.094 | 0.086 | 0.127 |  | 0.154 | 0.131 | 0.16 | 0.18 | 0.131 | 0.075 | 0.173 |
| **RF** | 0.075 | 0.074 | 0.062 | 0.066 | 0.055 | 0.065 | 0.062 | 0.073 | 0.025 | 0.064 | 0.09 | 0.083 |  | 0.151 | 0.098 | 0.119 | 0.096 | 0.102 | 0.151 |
| **RG** | 0.015 | 0.026 | 0.064 | 0.066 | 0.063 | 0.059 | 0.057 | 0.024 | 0.082 | 0.063 | 0.098 | 0.075 | 0.066 |  | 0.118 | 0.118 | 0.141 | 0.09 | 0.068 |
| **RW** | 0.058 | 0.074 | 0.06 | 0.059 | 0.056 | 0.08 | 0.05 | 0.075 | 0.063 | 0.06 | 0.075 | 0.078 | 0.053 | 0.057 |  | 0.099 | 0.109 | 0.097 | 0.13 |
| **SP** | 0.065 | 0.064 | 0.046 | 0.045 | 0.072 | 0.065 | 0.069 | 0.069 | 0.074 | 0.052 | 0.072 | 0.095 | 0.054 | 0.06 | 0.043 |  | 0.103 | 0.097 | 0.137 |
| **SW** | 0.079 | 0.069 | 0.057 | 0.069 | 0.055 | 0.072 | 0.069 | 0.079 | 0.061 | 0.069 | 0.106 | 0.071 | 0.052 | 0.066 | 0.054 | 0.056 |  | 0.078 | 0.126 |
| **TAU** | 0.068 | 0.062 | 0.058 | 0.061 | 0.038 | 0.083 | 0.072 | 0.066 | 0.067 | 0.061 | 0.088 | 0.051 | 0.051 | 0.054 | 0.045 | 0.048 | 0.047 |  | 0.135 |
| **WW** | 0.044 | 0.052 | 0.08 | 0.068 | 0.073 | 0.048 | 0.053 | 0.049 | 0.084 | 0.06 | 0.092 | 0.088 | 0.071 | 0.033 | 0.055 | 0.065 | 0.058 | 0.064 |  |

**Table 2B Table:** **Fst values (below the diagonal) and Jost's D values (above the diagonal) for the North Rhine-Westphalian AMUs.**

|  | **BB** | **EB** | **EFW** | **EG** | **EHU** | **ENP** | **EZM** | **MI** | **MOA** | **MOB** | **NR** | **NS** | **RK** | **SE** | **SIO** | **SIW** | **UF** | **WB** | **WGS** | **WH** |
| --- | --- | --- | --- | --- | --- | --- | --- | --- | --- | --- | --- | --- | --- | --- | --- | --- | --- | --- | --- | --- |
| **BB** |  | 0.178 | 0.156 | 0.113 | 0.239 | 0.226 | 0.236 | 0.202 | 0.149 | 0.055 | 0.237 | 0.231 | 0.315 | 0.24 | 0.034 | 0.074 | 0.244 | 0.061 | 0.072 | 0.249 |
| **EB** | 0.077 |  | 0.288 | 0.278 | 0.318 | 0.309 | 0.324 | 0.335 | 0.266 | 0.21 | 0.317 | 0.305 | 0.378 | 0.365 | 0.163 | 0.246 | 0.248 | 0.191 | 0.172 | 0.347 |
| **EFW** | 0.053 | 0.095 |  | 0.188 | 0.058 | 0.066 | 0.031 | 0.271 | 0.198 | 0.16 | 0.348 | 0.203 | 0.414 | 0.305 | 0.174 | 0.204 | 0.295 | 0.155 | 0.18 | 0.249 |
| **EG** | 0.056 | 0.109 | 0.074 |  | 0.239 | 0.228 | 0.268 | 0.246 | 0.116 | 0.086 | 0.244 | 0.35 | 0.311 | 0.151 | 0.109 | 0.169 | 0.239 | 0.149 | 0.116 | 0.252 |
| **EHU** | 0.083 | 0.114 | 0.024 | 0.1 |  | 0.023 | 0.027 | 0.315 | 0.268 | 0.237 | 0.39 | 0.286 | 0.488 | 0.336 | 0.255 | 0.293 | 0.352 | 0.239 | 0.272 | 0.279 |
| **ENP** | 0.077 | 0.105 | 0.026 | 0.095 | 0.015 |  | 0.019 | 0.286 | 0.269 | 0.209 | 0.424 | 0.295 | 0.5 | 0.328 | 0.24 | 0.258 | 0.374 | 0.225 | 0.245 | 0.253 |
| **EZM** | 0.073 | 0.104 | 0.015 | 0.098 | 0.016 | 0.009 |  | 0.261 | 0.306 | 0.205 | 0.402 | 0.253 | 0.482 | 0.39 | 0.259 | 0.255 | 0.371 | 0.21 | 0.242 | 0.235 |
| **MI** | 0.104 | 0.166 | 0.109 | 0.12 | 0.134 | 0.122 | 0.119 |  | 0.315 | 0.217 | 0.248 | 0.301 | 0.433 | 0.282 | 0.203 | 0.206 | 0.231 | 0.24 | 0.212 | 0.334 |
| **MOA** | 0.058 | 0.106 | 0.082 | 0.047 | 0.121 | 0.115 | 0.113 | 0.141 |  | 0.067 | 0.279 | 0.307 | 0.275 | 0.195 | 0.139 | 0.205 | 0.249 | 0.152 | 0.147 | 0.264 |
| **MOB** | 0.022 | 0.071 | 0.054 | 0.038 | 0.083 | 0.076 | 0.066 | 0.106 | 0.032 |  | 0.251 | 0.217 | 0.271 | 0.22 | 0.057 | 0.098 | 0.242 | 0.054 | 0.068 | 0.256 |
| **NR** | 0.113 | 0.145 | 0.136 | 0.11 | 0.165 | 0.162 | 0.16 | 0.179 | 0.123 | 0.105 |  | 0.394 | 0.26 | 0.367 | 0.242 | 0.273 | 0.062 | 0.259 | 0.285 | 0.363 |
| **NS** | 0.099 | 0.128 | 0.089 | 0.143 | 0.122 | 0.123 | 0.109 | 0.168 | 0.134 | 0.096 | 0.176 |  | 0.45 | 0.342 | 0.148 | 0.138 | 0.422 | 0.211 | 0.153 | 0.285 |
| **RK** | 0.154 | 0.185 | 0.188 | 0.159 | 0.207 | 0.201 | 0.199 | 0.243 | 0.162 | 0.141 | 0.164 | 0.248 |  | 0.296 | 0.334 | 0.351 | 0.274 | 0.379 | 0.332 | 0.416 |
| **SE** | 0.095 | 0.14 | 0.108 | 0.069 | 0.128 | 0.127 | 0.131 | 0.144 | 0.086 | 0.078 | 0.154 | 0.153 | 0.17 |  | 0.258 | 0.261 | 0.339 | 0.318 | 0.222 | 0.283 |
| **SIO** | 0.021 | 0.075 | 0.058 | 0.056 | 0.087 | 0.078 | 0.077 | 0.092 | 0.057 | 0.027 | 0.112 | 0.083 | 0.163 | 0.093 |  | 0.033 | 0.204 | 0.077 | 0.013 | 0.275 |
| **SIW** | 0.039 | 0.088 | 0.067 | 0.076 | 0.096 | 0.085 | 0.081 | 0.094 | 0.078 | 0.042 | 0.119 | 0.082 | 0.183 | 0.105 | 0.017 |  | 0.227 | 0.113 | 0.044 | 0.272 |
| **UF** | 0.107 | 0.126 | 0.119 | 0.105 | 0.155 | 0.148 | 0.148 | 0.161 | 0.112 | 0.101 | 0.051 | 0.186 | 0.163 | 0.144 | 0.095 | 0.101 |  | 0.234 | 0.242 | 0.395 |
| **WB** | 0.029 | 0.078 | 0.059 | 0.07 | 0.089 | 0.082 | 0.072 | 0.119 | 0.072 | 0.034 | 0.128 | 0.108 | 0.192 | 0.114 | 0.033 | 0.047 | 0.117 |  | 0.095 | 0.282 |
| **WGS** | 0.029 | 0.073 | 0.057 | 0.052 | 0.088 | 0.079 | 0.074 | 0.093 | 0.055 | 0.023 | 0.115 | 0.079 | 0.159 | 0.093 | 0.008 | 0.019 | 0.097 | 0.036 |  | 0.264 |
| **WH** | 0.097 | 0.13 | 0.101 | 0.099 | 0.124 | 0.115 | 0.106 | 0.158 | 0.11 | 0.094 | 0.157 | 0.133 | 0.212 | 0.126 | 0.097 | 0.101 | 0.151 | 0.109 | 0.093 |  |

**Table 2C Table:** **Fst values for the comparison of North Rhine-Westphalian AMUs (rows) with Hessian AMUs (columns).**

|  | **BKW** | **DB** | **GF** | **HV** | **HW** | **KF** | **KNU** | **LB** | **MKW** | **NV** | **OD** | **PL** | **RF** | **RG** | **RW** | **SP** | **SW** | **TAU** | **WW** |
| --- | --- | --- | --- | --- | --- | --- | --- | --- | --- | --- | --- | --- | --- | --- | --- | --- | --- | --- | --- |
| **BB** | 0.099 | 0.068 | 0.132 | 0.159 | 0.11 | 0.151 | 0.138 | 0.113 | 0.234 | 0.142 | 0.194 | 0.157 | 0.176 | 0.05 | 0.122 | 0.123 | 0.164 | 0.098 | 0.117 |
| **EB** | 0.082 | 0.101 | 0.339 | 0.281 | 0.29 | 0.277 | 0.26 | 0.217 | 0.312 | 0.3 | 0.431 | 0.348 | 0.285 | 0.206 | 0.307 | 0.303 | 0.346 | 0.311 | 0.232 |
| **EFW** | 0.061 | 0.074 | 0.2 | 0.198 | 0.177 | 0.245 | 0.24 | 0.208 | 0.268 | 0.21 | 0.227 | 0.149 | 0.216 | 0.163 | 0.198 | 0.23 | 0.204 | 0.158 | 0.183 |
| **EG** | 0.068 | 0.086 | 0.229 | 0.195 | 0.195 | 0.199 | 0.204 | 0.209 | 0.181 | 0.239 | 0.273 | 0.223 | 0.186 | 0.145 | 0.141 | 0.168 | 0.163 | 0.16 | 0.138 |
| **EHU** | 0.088 | 0.107 | 0.274 | 0.276 | 0.245 | 0.316 | 0.281 | 0.295 | 0.366 | 0.302 | 0.326 | 0.242 | 0.288 | 0.233 | 0.261 | 0.332 | 0.282 | 0.227 | 0.25 |
| **ENP** | 0.081 | 0.096 | 0.217 | 0.256 | 0.217 | 0.276 | 0.243 | 0.255 | 0.298 | 0.263 | 0.317 | 0.215 | 0.249 | 0.216 | 0.246 | 0.292 | 0.242 | 0.213 | 0.243 |
| **EZM** | 0.078 | 0.095 | 0.253 | 0.284 | 0.236 | 0.309 | 0.267 | 0.268 | 0.314 | 0.291 | 0.258 | 0.208 | 0.269 | 0.221 | 0.276 | 0.306 | 0.286 | 0.231 | 0.261 |
| **MI** | 0.126 | 0.11 | 0.144 | 0.135 | 0.134 | 0.156 | 0.136 | 0.127 | 0.231 | 0.279 | 0.277 | 0.284 | 0.208 | 0.271 | 0.222 | 0.258 | 0.284 | 0.247 | 0.267 |
| **MOA** | 0.071 | 0.077 | 0.116 | 0.093 | 0.1 | 0.1 | 0.081 | 0.077 | 0.093 | 0.228 | 0.338 | 0.252 | 0.212 | 0.146 | 0.166 | 0.204 | 0.205 | 0.217 | 0.104 |
| **MOB** | 0.038 | 0.048 | 0.071 | 0.062 | 0.07 | 0.064 | 0.05 | 0.05 | 0.082 | 0.168 | 0.251 | 0.189 | 0.166 | 0.056 | 0.109 | 0.131 | 0.178 | 0.125 | 0.084 |
| **NR** | 0.127 | 0.132 | 0.15 | 0.124 | 0.169 | 0.153 | 0.162 | 0.123 | 0.129 | 0.311 | 0.29 | 0.417 | 0.23 | 0.243 | 0.285 | 0.261 | 0.261 | 0.26 | 0.267 |
| **NS** | 0.113 | 0.091 | 0.127 | 0.118 | 0.136 | 0.117 | 0.126 | 0.104 | 0.14 | 0.195 | 0.295 | 0.291 | 0.248 | 0.194 | 0.273 | 0.206 | 0.318 | 0.252 | 0.246 |
| **RK** | 0.199 | 0.189 | 0.224 | 0.213 | 0.222 | 0.209 | 0.203 | 0.193 | 0.199 | 0.216 | 0.24 | 0.252 | 0.179 | 0.198 | 0.364 | 0.327 | 0.381 | 0.41 | 0.335 |
| **SE** | 0.116 | 0.132 | 0.133 | 0.109 | 0.123 | 0.129 | 0.111 | 0.107 | 0.125 | 0.116 | 0.149 | 0.135 | 0.105 | 0.115 | 0.096 | 0.269 | 0.261 | 0.271 | 0.255 |
| **SIO** | 0.038 | 0.014 | 0.064 | 0.062 | 0.07 | 0.059 | 0.065 | 0.026 | 0.078 | 0.061 | 0.107 | 0.081 | 0.065 | 0.022 | 0.058 | 0.128 | 0.129 | 0.114 | 0.099 |
| **SIW** | 0.049 | 0.028 | 0.062 | 0.052 | 0.08 | 0.068 | 0.075 | 0.04 | 0.094 | 0.057 | 0.113 | 0.096 | 0.069 | 0.04 | 0.06 | 0.089 | 0.172 | 0.145 | 0.161 |
| **UF** | 0.113 | 0.111 | 0.145 | 0.121 | 0.168 | 0.135 | 0.146 | 0.097 | 0.124 | 0.129 | 0.157 | 0.18 | 0.115 | 0.113 | 0.129 | 0.123 | 0.126 | 0.136 | 0.22 |
| **WB** | 0.03 | 0.045 | 0.072 | 0.067 | 0.058 | 0.071 | 0.057 | 0.042 | 0.086 | 0.064 | 0.1 | 0.079 | 0.063 | 0.012 | 0.063 | 0.064 | 0.073 | 0.06 | 0.08 |
| **WGS** | 0.033 | 0.016 | 0.073 | 0.065 | 0.078 | 0.056 | 0.059 | 0.033 | 0.075 | 0.067 | 0.096 | 0.086 | 0.062 | 0.027 | 0.056 | 0.056 | 0.071 | 0.055 | 0.11 |
| **WH** | 0.109 | 0.117 | 0.12 | 0.111 | 0.127 | 0.13 | 0.119 | 0.108 | 0.122 | 0.12 | 0.132 | 0.15 | 0.107 | 0.111 | 0.118 | 0.102 | 0.118 | 0.111 | 0.236 |

**Table 2D Table:** **Jost's D values for the comparison of North Rhine-Westphalian AMUs (rows) with Hessian AMUs (columns).**

|  | **BKW** | **DB** | **GF** | **HV** | **HW** | **KF** | **KNU** | **LB** | **MKW** | **NV** | **OD** | **PL** | **RF** | **RG** | **RW** | **SP** | **SW** | **TAU** | **WW** |
| --- | --- | --- | --- | --- | --- | --- | --- | --- | --- | --- | --- | --- | --- | --- | --- | --- | --- | --- | --- |
| **BB** | 0.039 | 0.038 | 0.067 | 0.066 | 0.055 | 0.066 | 0.058 | 0.05 | 0.089 | 0.066 | 0.09 | 0.079 | 0.064 | 0.024 | 0.055 | 0.057 | 0.064 | 0.052 | 0.043 |
| **EB** | 0.196 | 0.222 | 0.137 | 0.113 | 0.144 | 0.118 | 0.1 | 0.095 | 0.142 | 0.121 | 0.174 | 0.164 | 0.121 | 0.085 | 0.126 | 0.119 | 0.147 | 0.136 | 0.09 |
| **EFW** | 0.144 | 0.202 | 0.087 | 0.078 | 0.079 | 0.09 | 0.079 | 0.08 | 0.102 | 0.082 | 0.105 | 0.082 | 0.081 | 0.063 | 0.075 | 0.081 | 0.081 | 0.074 | 0.072 |
| **EG** | 0.114 | 0.176 | 0.104 | 0.08 | 0.095 | 0.106 | 0.085 | 0.082 | 0.083 | 0.093 | 0.116 | 0.11 | 0.072 | 0.066 | 0.054 | 0.082 | 0.074 | 0.069 | 0.066 |
| **EHU** | 0.196 | 0.275 | 0.119 | 0.115 | 0.114 | 0.124 | 0.111 | 0.114 | 0.141 | 0.125 | 0.147 | 0.121 | 0.116 | 0.097 | 0.111 | 0.118 | 0.118 | 0.106 | 0.11 |
| **ENP** | 0.184 | 0.256 | 0.103 | 0.102 | 0.099 | 0.112 | 0.096 | 0.102 | 0.123 | 0.11 | 0.138 | 0.101 | 0.106 | 0.089 | 0.1 | 0.108 | 0.105 | 0.093 | 0.1 |
| **EZM** | 0.205 | 0.264 | 0.103 | 0.104 | 0.101 | 0.112 | 0.095 | 0.099 | 0.124 | 0.108 | 0.127 | 0.102 | 0.101 | 0.082 | 0.102 | 0.105 | 0.11 | 0.095 | 0.099 |
| **MI** | 0.249 | 0.221 | 0.283 | 0.258 | 0.276 | 0.292 | 0.278 | 0.262 | 0.129 | 0.139 | 0.16 | 0.147 | 0.118 | 0.123 | 0.118 | 0.129 | 0.134 | 0.122 | 0.138 |
| **MOA** | 0.141 | 0.195 | 0.251 | 0.201 | 0.235 | 0.224 | 0.186 | 0.178 | 0.222 | 0.097 | 0.131 | 0.115 | 0.081 | 0.066 | 0.071 | 0.094 | 0.081 | 0.089 | 0.056 |
| **MOB** | 0.071 | 0.118 | 0.155 | 0.165 | 0.17 | 0.141 | 0.137 | 0.095 | 0.216 | 0.064 | 0.096 | 0.085 | 0.059 | 0.033 | 0.044 | 0.059 | 0.068 | 0.06 | 0.04 |
| **NR** | 0.268 | 0.282 | 0.326 | 0.242 | 0.325 | 0.326 | 0.396 | 0.278 | 0.234 | 0.135 | 0.157 | 0.198 | 0.118 | 0.122 | 0.132 | 0.122 | 0.138 | 0.139 | 0.117 |
| **NS** | 0.225 | 0.143 | 0.223 | 0.236 | 0.288 | 0.218 | 0.316 | 0.144 | 0.295 | 0.111 | 0.155 | 0.155 | 0.114 | 0.102 | 0.118 | 0.103 | 0.145 | 0.118 | 0.117 |
| **RK** | 0.37 | 0.376 | 0.403 | 0.366 | 0.407 | 0.4 | 0.422 | 0.377 | 0.327 | 0.407 | 0.471 | 0.456 | 0.316 | 0.393 | 0.199 | 0.195 | 0.202 | 0.214 | 0.185 |
| **SE** | 0.269 | 0.336 | 0.318 | 0.259 | 0.252 | 0.289 | 0.225 | 0.255 | 0.274 | 0.276 | 0.329 | 0.29 | 0.244 | 0.317 | 0.205 | 0.111 | 0.11 | 0.113 | 0.104 |
| **SIO** | 0.077 | 0.028 | 0.134 | 0.13 | 0.141 | 0.124 | 0.166 | 0.061 | 0.182 | 0.132 | 0.248 | 0.163 | 0.151 | 0.052 | 0.137 | 0.056 | 0.059 | 0.055 | 0.045 |
| **SIW** | 0.111 | 0.053 | 0.122 | 0.101 | 0.168 | 0.132 | 0.188 | 0.089 | 0.221 | 0.124 | 0.27 | 0.188 | 0.162 | 0.102 | 0.112 | 0.046 | 0.08 | 0.063 | 0.063 |
| **UF** | 0.225 | 0.224 | 0.327 | 0.247 | 0.344 | 0.263 | 0.359 | 0.216 | 0.232 | 0.302 | 0.324 | 0.38 | 0.235 | 0.227 | 0.291 | 0.256 | 0.25 | 0.275 | 0.101 |
| **WB** | 0.059 | 0.093 | 0.143 | 0.147 | 0.123 | 0.148 | 0.153 | 0.09 | 0.185 | 0.144 | 0.219 | 0.142 | 0.133 | 0.017 | 0.153 | 0.133 | 0.146 | 0.105 | 0.04 |
| **WGS** | 0.083 | 0.03 | 0.176 | 0.167 | 0.186 | 0.124 | 0.146 | 0.064 | 0.188 | 0.172 | 0.251 | 0.189 | 0.152 | 0.057 | 0.136 | 0.152 | 0.18 | 0.117 | 0.043 |
| **WH** | 0.257 | 0.288 | 0.255 | 0.222 | 0.311 | 0.297 | 0.326 | 0.25 | 0.285 | 0.255 | 0.252 | 0.328 | 0.259 | 0.275 | 0.284 | 0.237 | 0.263 | 0.244 | 0.102 |


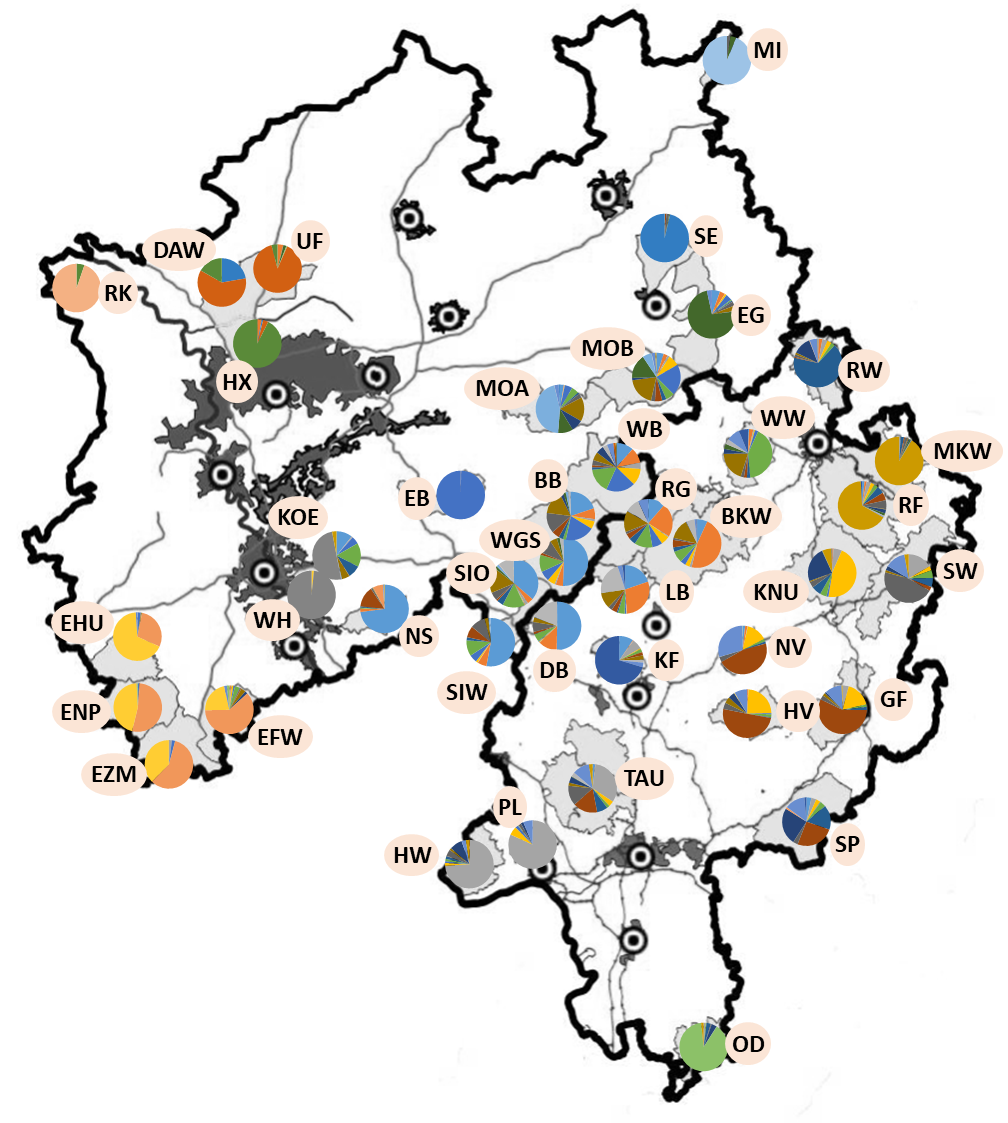


**Fig 1.** **Distribution of Clusters according to DAPC.** DAW = 9 samples; KOE = 4 samples. The map was constructed in QGIS, Version 3.28.3 [71]. The free and open source public software was downloaded from <http://qgis.org>, including the license terms (http://qgis.org/license).


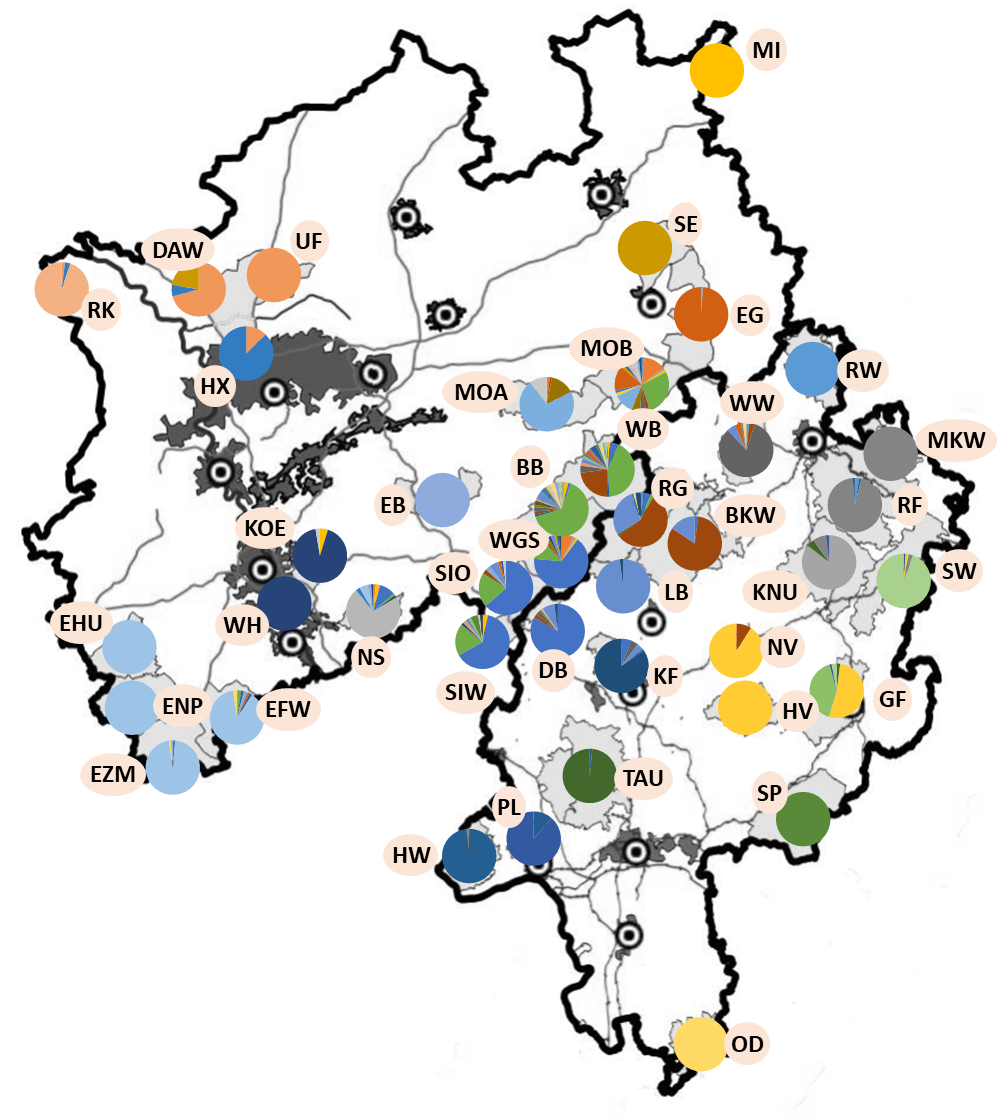


**Fig 2.** **Distribution of Clusters according to BAPS.** DAW = 9 samples; KOE = 4 samples.


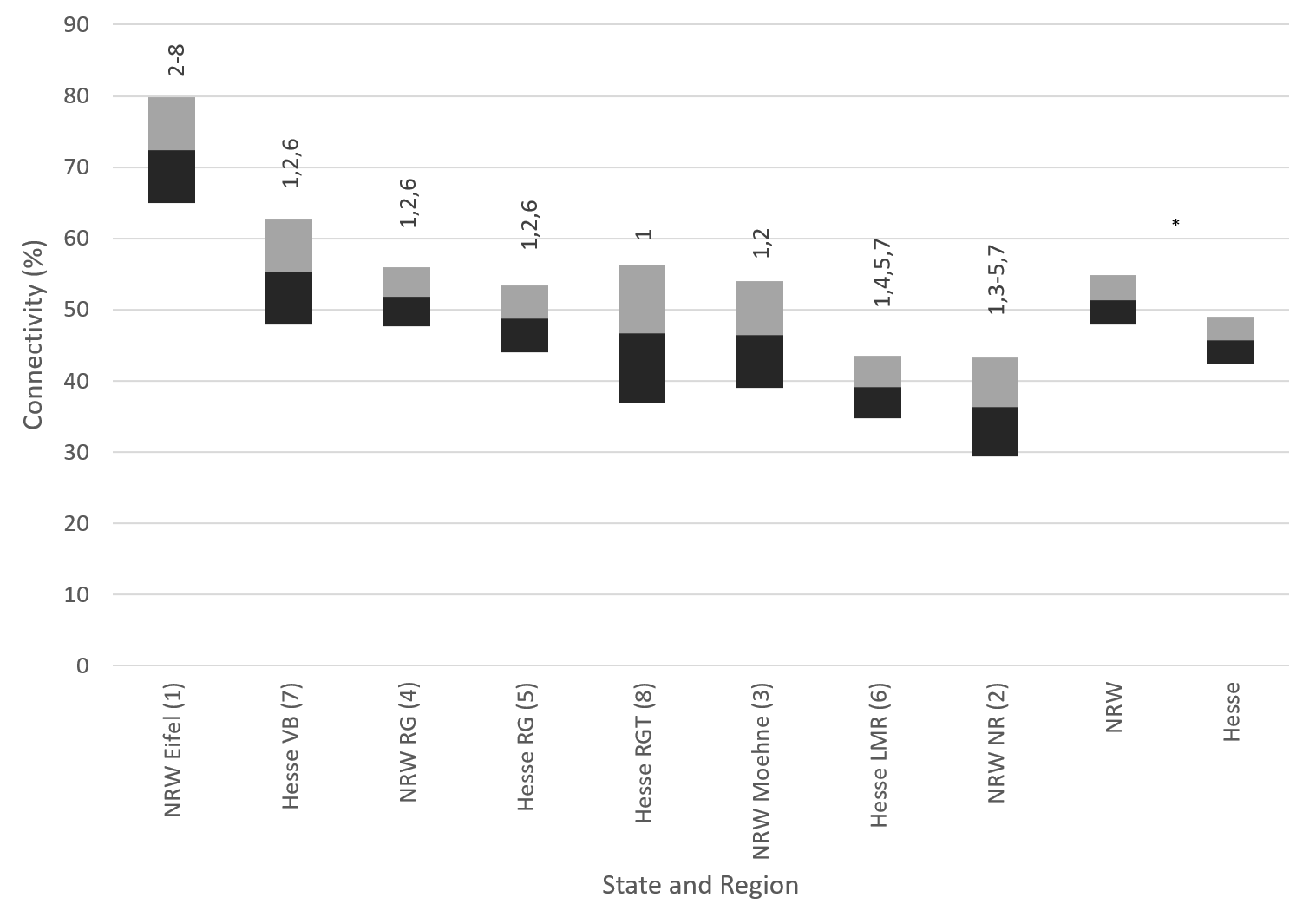


**Fig 3. Mean connectivity of AMUs within regions and states with neighbouring AMUs at a maximum geographical distance between AMUs of 40 km (average 28.2 km).** See Table 1 for abbreviations. Dark grey: lower 95% confidence interval, light grey: upper 95% confidence interval. Transition: mean value; * AMUs in NRW and Hesse are significantly different (P<0.05); superscript numbers: AMUs with significantly different connectivity.


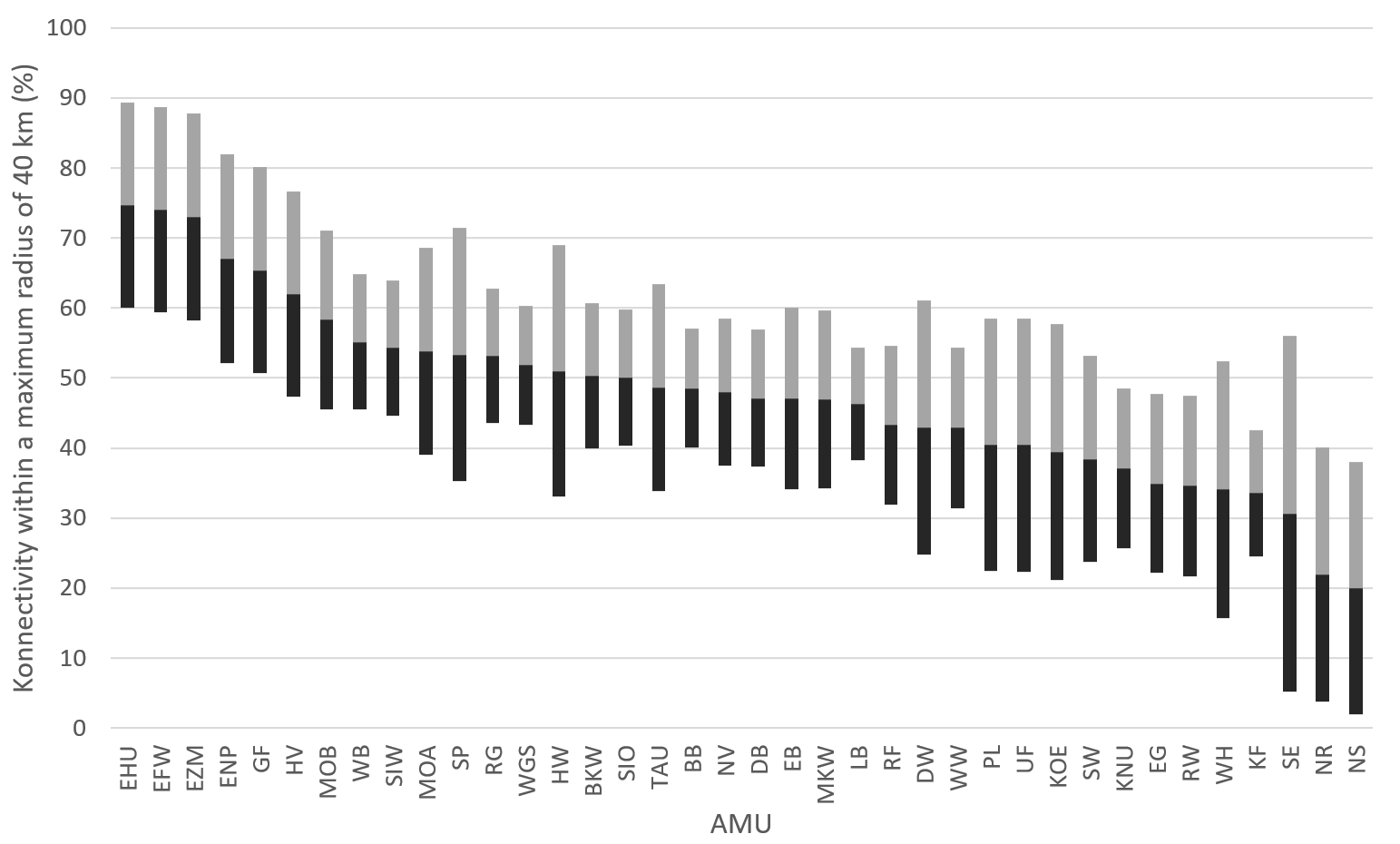


**Fig 4 Fig. Mean connectivity of AMUs with neighbouring AMUs at a maximum geographical distance between AMUs of 40 km (average 28.2 km).** See Table 1 for abbreviations. Dark grey: lower 95% confidence interval, light grey: upper 95% confidence interval. Transition: mean value.
